# Differentially-expressed mRNAs, microRNAs and long noncoding RNAs in intervertebral disc degeneration identified by RNA-sequencing

**DOI:** 10.1080/21655979.2021.1899533

**Published:** 2021-03-25

**Authors:** Zhimin Li, Yu Sun, Maolin He, Jianwei Liu

**Affiliations:** a Department of Spine Osteopathia, The First Affiliated Hospital of Guangxi Medical University, Nanning Guangxi, P.R China; b Department of Osteology, The Third Affiliated Hospital of Guangxi Medical University, Nanning Guangxi, P.R China

**Keywords:** Intervertebral disc degeneration, rna-sequencing, lncRNA xist, hsa-miR-4775, hsa-miR-424-5p, pla2g7, amot, tgfbr3

## Abstract

The underlying molecular mechanisms of intervertebral disc degeneration (IDD) remain unclear. This study aimed to identify the crucial molecules and explore the function of noncoding RNAs and related pathways in IDD. We randomly selected three samples each from an IDD and a spinal cord injury group (control) for RNA-sequencing. We identified 463 differentially-expressed long noncoding RNAs (lncRNAs), 47 differentially-expressed microRNAs (miRNAs), and 1,334 differentially-expressed mRNAs in IDD. Three hundred fifty-eight lncRNAs as cis-regulators could potentially target 865 genes. Protein–protein interaction (PPI) network analysis confirmed that *IL-6, VEGFA, IGF1, MMP9, CXCL8, FGF2, IL1B, CCND1, ITGAM, PTPRC, FOS* and *PTGS2* were hub genes. We built a competing endogenous RNA (ceRNA) network and identified lncRNA XIST–hsa-miR-4775–PLA2G7 and lncRNA XIST–hsa-miR-424-5p–AMOT/TGFBR3 ceRNA axes. Quantitative real-time PCR (qRT-PCR) was implemented in 15 IDD samples and 15 controls to validate differentially-expressed genes in ceRNA axes. From the ceRNA network, gene ontology (GO) enrichment analysis indicated that noncoding RNAs were associated with several biological processes, including extracellular matrix organization, extracellular structure organization, leukocyte migration, and mesenchyme development. Kyoto Encyclopedia of Genes and Genomes (KEGG) pathway analysis revealed that noncoding RNAs were associated with several pathways including the AGE-RAGE signaling pathway, PI3K-Akt signaling pathway, axon guidance, and osteoclast differentiation. These results indicate that some specific noncoding RNAs and ceRNA axes may be vital during the development of IDD, and may have potential as alternative diagnostic biomarkers as well as novel therapeutic strategies for IDD.

## Graphical Abstract

**Figure:**
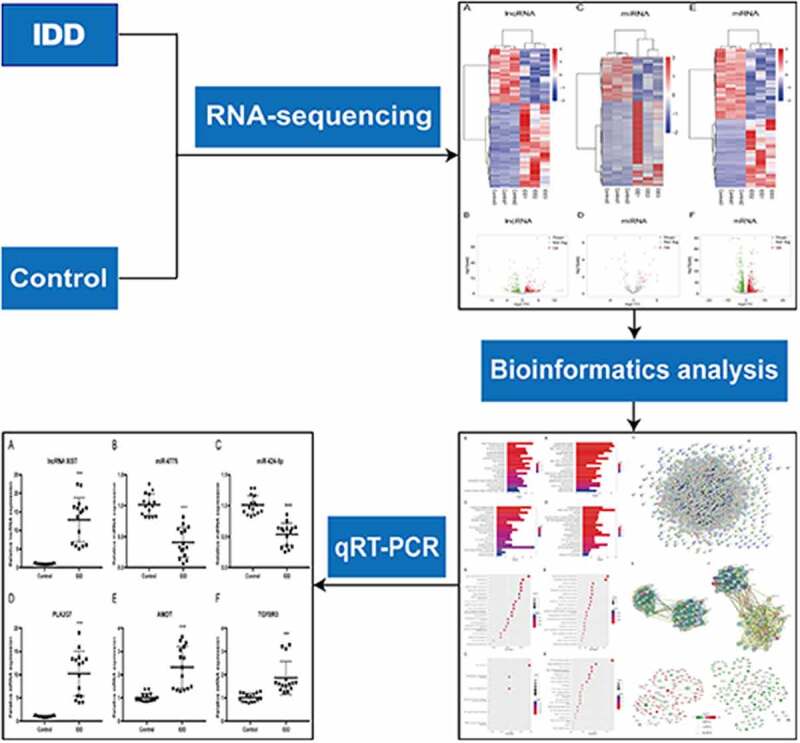

## Introduction

Intervertebral disc degeneration (IDD) is a major cause of neck and lower back pain, which results in disability and declining health and brings serious socioeconomic consequences around the world [1,2]. The available evidence proves that the occurrence of IDD is associated with age, but that many other factors contribute to the disease process, such as physical damage, genetic predisposition, apoptosis, microenvironmental changes, and inflammation [3]. At present, the main treatments for IDD are drugs and surgery, which can ease pain symptoms temporarily. However, these treatments cannot provide a permanent cure for the complex mechanisms underlying IDD [4,5]. Hence, it is essential to thoroughly explore the underlying molecular mechanisms of IDD, which will help to develop novel and promising strategies for its treatment.

Currently, available evidence indicates that microRNAs (miRNAs/miRs) and long noncoding RNAs (lncRNAs) are vital modulators in the initiation and progression of IDD [6]. MiRNAs are a type of short non-coding RNA molecule of approximately 20–22 nucleotides [7]. They can trigger either translation inhibition or mRNA degradation when they bind to the 3ʹ-untranslated region of their target mRNAs [8]. LncRNAs are a kind of non-coding RNA more than 200 nucleotides in length. As miRNA ‘sponges’, they may competitively bind to miRNAs and then regulate their target genes [9]. Consequently, lncRNAs may be involved in a series of biological processes, including transcription, splicing and translation [10]. Therefore, lncRNA–miRNA–mRNA interactions may contribute to the pathological process of IDD. Recently, Zhu et al. [11] elucidated the interactions of mRNA, miRNA, and lncRNA in the human lumbar disc by analyzing a series of public datasets. However, the microarray analysis method may produce biased results [12], while RNA sequencing (RNA-seq) is independently applied to genome annotation or sequences. In addition, RNA-seq can detect previously unknown miRNAs and lncRNAs without bias [13]. Moreover, the aforementioned study was lacking in further experimental verification of the findings of bioinformatics analysis. Recently, Zhao et al. [14] identified differentially-expressed lncRNAs (DELs) and differentially-expressed mRNAs (DEMs) by RNA-seq in IDD, but did not identify differentially-expressed miRNAs (DEMis). As far as we know, there have been few reported studies to date exploring the regulatory mechanisms of lncRNA–miRNA–mRNA using RNA-seq by constructing a ceRNA network for IDD.

This study was designed to identify DELs, DEMis and DEMs between an IDD group and a control (spinal cord injury) group by RNA-seq. Subsequently, a ceRNA network was established to reveal the underlying function of noncoding RNAs in IDD. Furthermore, two novel ceRNA axes, lncRNA XIST–hsa-miR-4775–PLA2G7 and lncRNA XIST–hsa-miR-424-5p–AMOT/TGFBR3 were identified and validated by quantitative real-time PCR (qRT-PCR). The results may reveal alternative diagnostic biomarkers as well as novel therapeutic strategies for IDD.

## Materials and Methods

### Tissue sample collection

Samples of nucleus pulposus (NP) tissue were obtained from IDD patients (n = 15, mean 61.9 age) and those with spinal cord injury as controls (n = 15, mean 34.5 age) (Table S1). According to the Pfirrmann grading classification, magnetic resonance imaging scans were used to assess the degree of disc degeneration [15]. All patients had undergone surgery in The Third Affiliated Hospital of Guangxi Medical University, Nanning, Guangxi, P.R China. The study received approval from the Hospital Ethics Committee and followed the Ethical Principles outlined in the declaration of Helsinki [16]. Informed consent was obtained from each patient in this study.

### RNA extraction

Total RNA from the NP of both groups was isolated using TRIzol reagent (Invitrogen, Carlsbad, CA, USA) according to the standard operating procedure. The RNA samples were treated with DNase I to remove residual genomic DNA. Degradation and contamination of RNA were assessed by 1% agarose gel electrophoresis. The purity of RNA was measured using a NanoPhotometer® spectrophotometer (Implen, Calabasas, CA, USA). RNA was quantified using a Qubit® 2.0 Fluorometer (Life Technologies, Carlsbad, CA, USA). RNA quality was analyzed using the Bioanalyzer 2100 system (Agilent Technologies, Santa Clara, CA, USA). RNA-seq and subsequent experiments were conducted when the RNAs met the criteria (pure and intact RNAs).

### Library preparation, clustering and RNA-seq

All the RNA-seq was conducted by Anrenx Biotechnology, Nanning, China. RNA from three IDD patients and three controls was randomly selected for RNA-seq. A total of 3 μg of RNA from each sample was used to prepare lncRNA and mRNA sequencing libraries. Additionally, 1 μg of RNA from each sample was utilized for miRNA sequencing. We used the Epicenter Ribo-zero™ rRNA Removal Kit (Epicenter Inc., Madison, WI, USA) to eliminate ribosomal RNA. Following standard manufacturer’s protocols, lncRNA and mRNA libraries were obtained using a NEBNext® Ultra™ Directional RNA Library Prep Kit while miRNA libraries were constructed with a NEBNext® Multiplex Small RNA Library Prep Set for Illumina® (New England Biolabs (NEB), Ipswich, MA, USA). Based on the standard manufacturer’s protocols, the cluster analysis was conducted using cBot software with a TruSeq PE Cluster Kit v3-cBot-HS (Illumina Inc., San Diego, CA, USA). The generated cluster libraries were sequenced on an Illumina Hiseq 4000 platform (Illumina Inc., San Diego, CA, USA).

### RNA-Seq reads mapping and analysis of differentially-expressed genes (DEGs)

The clean data of lncRNAs, miRNAs and mRNAs were generated by eliminating low-quality reads from the raw data. The clean data were evaluated for a Phred quality value ≥ 20. All further analyses were performed using high-quality cleans. Clean reads from lncRNA and mRNA libraries were aligned to the human reference genome hg38 using STAR v2.7 while the small RNA tags were mapped in miRbase release 22.1 using Bowtie [17]. Non-coding transcripts were screened out by either or all of the four tools (CNCI, CPC, Pfam-scan, CPAT), which were used to distinguish lncRNA from mRNA. The Cufflinks (v2.1.1) package was applied to reconstruct the mapped lncRNA reads [18]. For lncRNA and mRNA sequencing, the counts of transcripts per million (TPM) were computed in each gene group using in-house Perl scripts. As a small RNA, the read counts of miRNA were calculated in reads per million. Particularly, low-expression genes were discarded and the threshold was the sum of read counts < 10. The DESeq2 R package (1.26.0) was used to analyze the DEGs between the IDD group and the controls. DEGs were regarded as significantly different when an adjusted *P* < 0.05. Genes were identified as exhibiting significant differential expression when an absolute value of log2 (fold change) (log2 FC) > 1.

### Functional enrichment analysis

Gene ontology (GO) and Kyoto Encyclopedia of Genes and Genomes (KEGG) enrichment analyses were performed using the clusterProfiler R (v3.13.0) package. GO analysis was performed to understand the bio-function of DEGs. KEGG enrichment analysis was used to reveal the significant pathways of DEGs. GO and KEGG terms with corrected *P* < 0.05 were considered significantly enriched by DEGs.

### *Protein–protein interaction*(PPI) *network*

The DEGs between the IDD group and the controls were mapped to STRING (version 10.0). The PPI network was established and module analysis was conducted using Cytoscape software (version 3.6.1). The topological features of the PPI network were analyzed, including degree centrality, betweenness centrality, and closeness centrality. Cytoscape software with the Molecular Complex Detection (MCODE; version:1.4.2) plug-in was applied to screen out key modules. Parameters were set with a degree cutoff of 2, node score cutoff of 0.2, k-core of 2 and a maximum depth of 100. GO and KEGG enrichment analyses were implemented in the modules.

### CeRNA network

Gene co-expression networks were established with the normalized signal intensity of DELs, DEMis and DEMs in the IDD group compared with the controls. lncRNA–miRNA, miRNA–mRNA, and lncRNA–mRNA interaction pairs were based on Pearson’s correlation coefficient value over 0.95. Moreover, the DEGs among the target genes were selected to conduct a network using Cytoscape software (V. 3.2.1).

### Validation by qRT-PCR

DELs, DEMis and DEMs in the ceRNA network from the RNA-seq results were validated by qRT-PCR using a LightCycler® 96 System (Roche, Basel, Switzerland). The primer sequences were designed using Primer 5.0 and listed in Table 1. U6 RNA acted as an internal control of miRNAs while glyceraldehyde-3-phosphate dehydrogenase (GAPDH) served as an internal mRNA or lncRNA control. The relative gene expression was analyzed using the 2− ΔΔCt method.

**Table 1.** Primers used in qRT-PCR

Primer	Sequence (5ʹ-3ʹ)	
GAPDH	Forward	5ʹ-GACAGTCAGCCGCATCTTCT-3’
	Reverse	5ʹ-GCGCCCAATACGACCAAATC-3’
U6	Forward	5ʹ-CTCGCTTCGGCAGCACA-3’
	Reverse	5ʹ-AACGCTTCACGAATTTGCGT-3’
lncRNA XIST	Forward	5ʹ-CCCTCATCCCCACTTTTCCC-3’
	Reverse	5ʹ-TGGAATGAGCAGTGTGCGAT-3’
miR-4775	Forward	5ʹ-TTAATTTTTTGTTTCGG-3’
	Reverse	5ʹ-CAGTGCGTGTCGTGGAGT-3’
miR-424-3p	Forward	5ʹ-CAGCAGCAATTCATGTT-3’
	Reverse	5ʹ-CAGTGCGTGTCGTGGAGT-3’
PLA2G7	Forward	5ʹ-CGCAGGGCATTGCCAACTAT-3’
	Reverse	5ʹ-TCCTTTCCGCAGACCTGATG-3’
AMOT	Forward	5ʹ-TTTGCCTTCAGGGAGCTGCTA-3’
	Reverse	5ʹ-GTCCAACCACTGGTCACTCT-3’
TGFBR3	Forward	5ʹ-TTTCCTCTTCCCAGCGAGTG-3’
	Reverse	5ʹ-TGCAATTTTCAAACTGCCTCT-3’

qRT-PCR, quantitative real-time polymerase chain reaction; lncRNA, long noncoding RNA; miR, microRNA.

### Statistical analysis

The data are presented as a mean ± standard deviation (SD). Experiments were carried out in triplicate. Differences between the two groups were analyzed by two-tailed Student’s t-test and one-way ANOVA with SPSS 22.0 software (SPSS Inc., Chicago, IL, USA). Differences were considered statistically significant when *P* < 0.05.

## Results

In this study, we explored in detail the underlying molecular mechanisms in IDD. We aimed to identify the new crucial molecules and explore the function of noncoding RNAs and related pathways in IDD. We identified the DELs, DEMis and DEMs in IDD by RNA-seq. In order to explore underlying molecular mechanisms, a ceRNA network was built and functional enrichment analyses were conducted. Further, qRT-PCR was implemented to validate the lncRNA XIST–hsa-miR-4775–PLA2G7 and lncRNA XIST–hsa-miR-424-5p–AMOT/TGFBR3 ceRNA axes. We speculated that these two ceRNA axes may be vital during the development of IDD, and may have potential as alternative diagnostic biomarkers as well as novel therapeutic targets for IDD.

### RNA-seq and transcriptome reconstruction

RNAs from the IDD and control groups were sequenced. In the lncRNA and mRNA library, 246,903,006 raw reads were generated from the IDD group libraries, while 255,980,110 raw reads were generated from the control libraries. We thus collected 502,883,116 clean reads in both libraries. First, the clean reads were mapped to the reference genome. Almost 90.46% of the reads in the control group mapped onto the reference genome while 99.07% were mapped in the IDD group (Table 2). In the miRNA library, 32,784,020 and 33,899,700 raw reads were collected from the IDD group and controls, respectively. In total, 65,250,196 clean reads were collected in both libraries. The obtained clean reads were aligned onto the reference genome sequence using Bowtie. Approximately 81.63% of the clean reads generated from the control group were miRNA mapped while 83.57% were mapped in the IDD group (Table 3).

**Table 2.** Summary of data from lncRNA-seq and mRNA-seq for the IDD group and controls

Sample	Raw reads	Clean reads	Total mapped	Unique mapped
Control	255,980,110	246,404,608 (100%)	222,904,726 (90.46%)	218,093,918 (88.51%)
IDD	246,903,006	242,152,674 (100%)	239,897,345 (99.07%)	234,926,662 (97.02%)

lncRNA-seq, long noncoding RNA sequencing; mRNA-seq, mRNA sequencing; IDD, intervertebral disc degeneration.

**Table 3.** Summary of data from miRNA-seq for the IDD group and controls

Sample	Raw reads	Clean reads	Total mapped	miRNA mapped
Control	33,899,700	33,470,810 (100%)	29,651,791 (88.59%)	27,323,338 (81.63%)
IDD	32,784,020	31,779,386 (100%)	29,608,854 (93.17%)	26,556,974 (83.57%)

miRNA-seq, microRNA sequencing; IDD, intervertebral disc degeneration; miRNA, microRNA.

### Analysis of DELs, DEMis and DEMs

**Figure 1.**
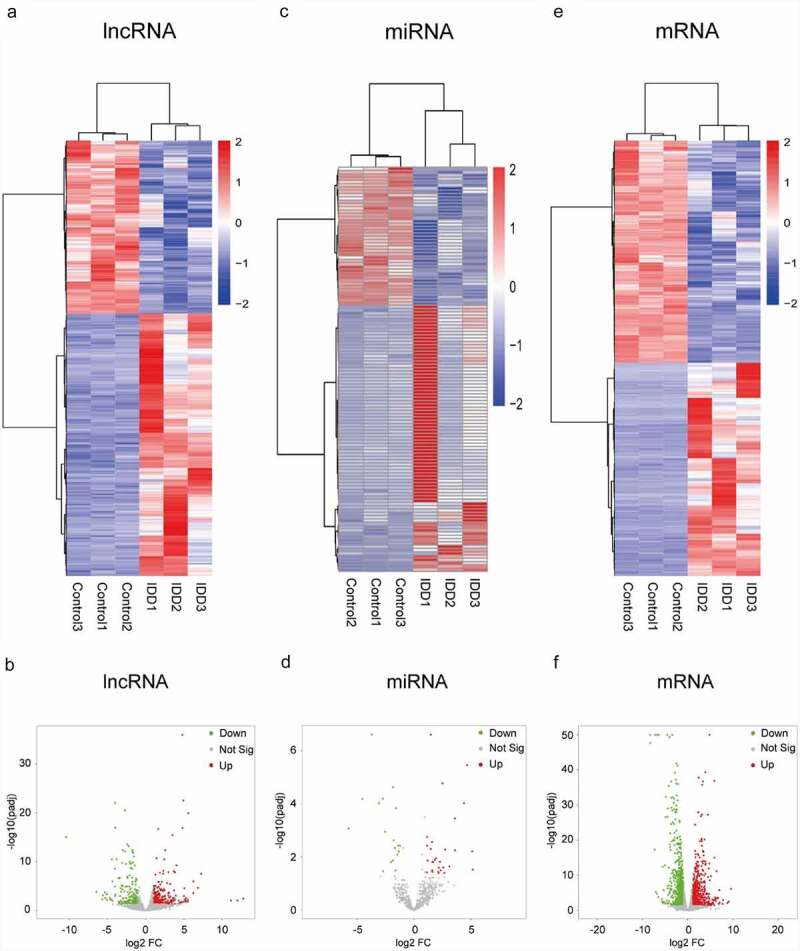
Expression profiles of lncRNAs, miRNAs and mRNAs. (a) Heat map of DELs in the IDD group compared with the control group. (b) Volcano plot of the DELs. (c) Heat map of DEMis. (d) Volcano plot of DEMis. (e) Heat map of DEMs. (f) Volcano plot of DEMs. IDD, intervertebral disc degeneration; lncRNA, long noncoding RNA; miRNA, microRNA; DELs, differentially-expressed lncRNAs; DEMis, differentially-expressed microRNAs; DEMs, differentially-expressed mRNAs

Levels of transcript expression were analyzed using Cufflinks software. The results of DEL, DEMi and DEM analyses are presented in heatmap and volcano plots. In the expression profile of lncRNAs (Figure 1a, B), a total of 463 DELs were detected. Among them, 279 lncRNAs were up-regulated and 184 were down-regulated in IDD. The Venn diagram for screening lncRNAs is shown in Figure S1. Moreover, in the expression profiles of miRNAs (Figure 1c and D), we identified 47 DEMis in the IDD group. Among them, 29 miRNAs were up-regulated and 18 miRNAs were down-regulated. Additionally, 1,334 mRNAs were significantly differentially expressed. Amongst these mRNAs, 652 mRNAs were up-regulated, whereas 682 mRNAs were down-regulated (Figure 1e, F). The differences of DELs, DEMis and DEMs were statistically significant (adjusted *P* < 0.05) with an absolute value of log2 FC > 1.

Further, the top ten upregulated and downregulated lncRNAs, miRNAs and mRNAs are shown in Tables S2–4. For lncRNAs, TCON_S00228970 (log2 FC = 12.79) was the most significantly up-regulated and TCONS_00132082 (log2 FC = −10.45) was the most significantly down-regulated. For miRNAs, hsa-miR-3622a-5p (log2 FC = 5.19) was the most significantly up-regulated while hsa-miR-4284 (log2 FC = −5.78) was the most significantly down-regulated. As for mRNAs, COL9A3 (log2 FC = 9.55) was the most significantly up-regulated while MMP1 (log2 FC = −8.33) was the most significantly down-regulated.

### Prediction of potential target genes of lncRNAs

As cis-regulators, lncRNAs target neighboring protein-coding genes [19]. We noted that 358 DELs were transcribed near (< 100 kb) a corresponding protein-coding gene, and 865 targets were identified by filtering the lncRNAs (Table S5). Furthermore, GO and KEGG enrichment analyses were implemented to identify the functions and related pathways of these target mRNAs. The enriched GO terms of mRNAs targeted by up-regulated DELs were related to the pattern specification process, anterior/posterior pattern specification and regionalization, while mRNAs targeted by down-regulated DELs were associated with interleukin-1 receptor binding, receptor–ligand activity, and growth factor receptor binding (Figure S2A, B). KEGG enrichment analysis showed that they were involved in the Notch signaling pathway, glycosaminoglycan biosynthesis, and the p53 signaling pathway corresponding to up-regulated lncRNAs whereas cytokine–cytokine receptor interaction, focal adhesion and the PI3K–Akt signaling pathway corresponded to down-regulated lncRNAs (Figure S2C,D).

### Functional enrichment analysis of DEMs in IDD

**Figure 2.**
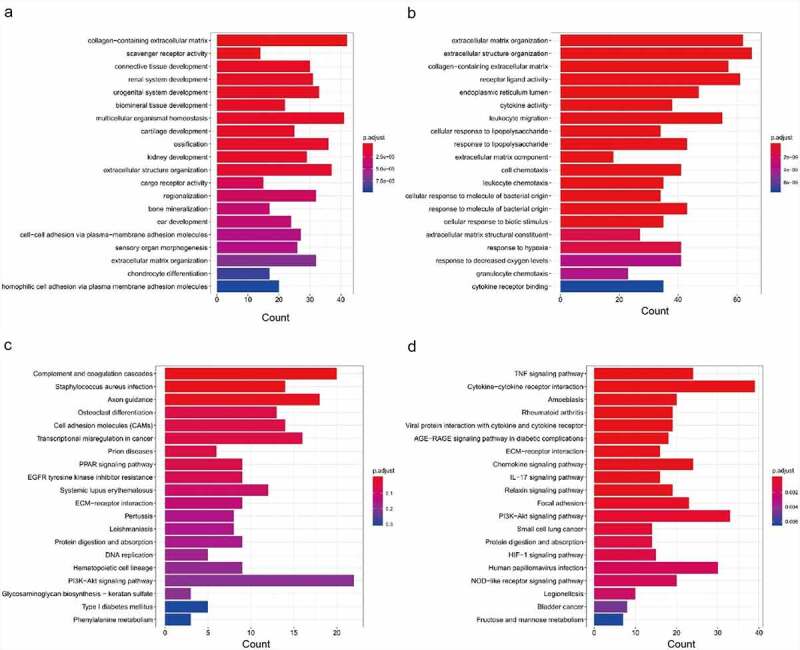
Functional enrichment analysis of differentially expressed mRNAs based on GO and KEGG. (a) GO analysis of up-regulated DEMs. (b) GO analysis of down-regulated DEMs. (c) KEGG pathway analysis of up-regulated DEMs. (d) KEGG pathway analysis of down-regulated DEMs. GO, Gene Ontology; DEMs, differentially-expressed mRNAs; KEGG, Kyoto Encyclopedia of Genes and Genomes

KEGG and GO analyses of DEMs were performed to illuminate the mechanisms involved in the pathological process of IDD. The top twenty GO terms of up-regulated DEMs are presented in Figure 2a. The top twenty GO terms of down-regulated DEMs are presented in Figure 2b. The enriched GO terms of up-regulated DEMs were closely related to collagen-containing extracellular matrix (ECM), scavenger receptor activity, and connective tissue development. Meanwhile, the enriched GO terms of down-regulated DEMs were closely related to ECM organization, extracellular structure organization, and collagen-containing ECM. The top twenty enriched KEGG pathways of up-regulated DEMs are presented in Figure 2c. The top twenty enriched KEGG pathways of down-regulated DEMs are presented in Figure 2d. The most enriched pathways were associated with complement and coagulation cascades, *Staphylococcus aureus* infection, and axon guidance in up-regulated DEMs. The most enriched pathways of down-regulated DEMs were connected with the TNF signaling pathway, cytokine−cytokine receptor interaction, and amoebiasis.

### PPI network

**Figure 3.**
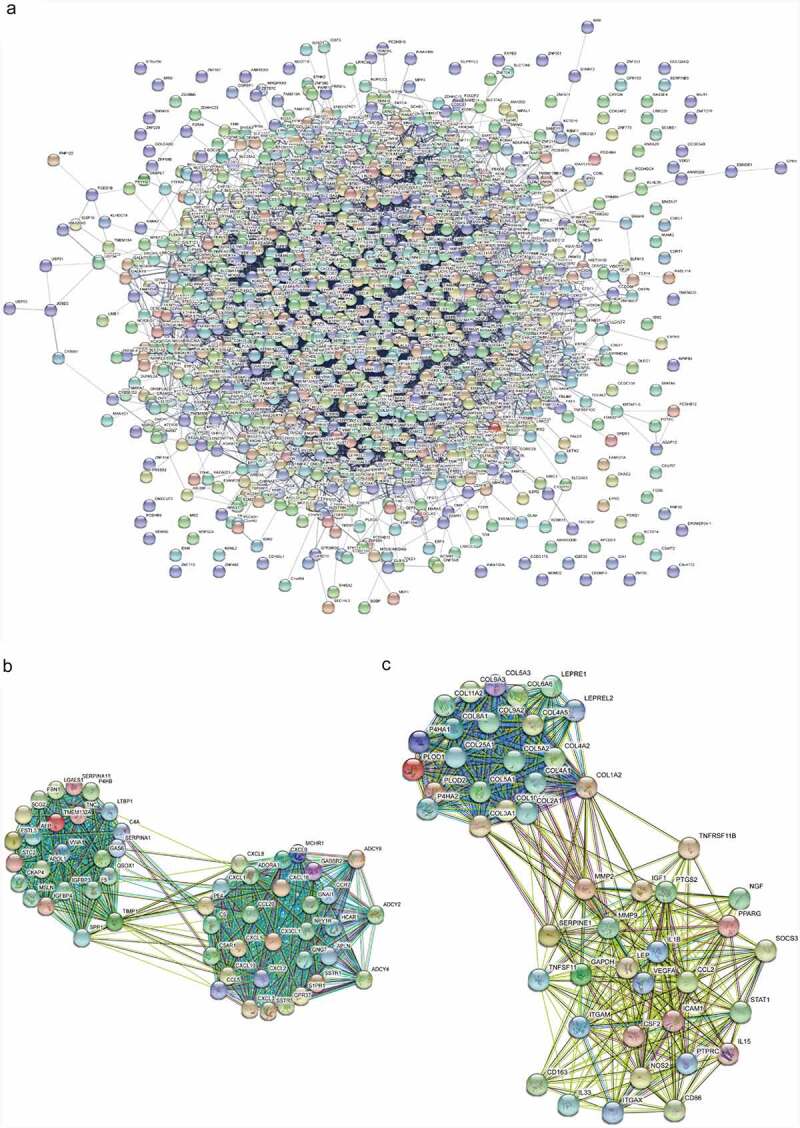
The protein-protein interaction network. (a) PPI network for the DEMs. (b) Sub-PPI network 1. (c) Sub-PPI network 2. PPI, protein-protein interaction; DEMs, differentially-expressed mRNAs

According to the DEMs, a PPI network was established which included 1,241 nodes and 9,949 interacting pairs (Figure 3a). IL6, VEGFA, IGF1, MMP9, CXCL8, FGF2, IL1B, CCND1, ITGAM, PTPRC, FOS and PTGS2 were considered hub genes in this PPI network (Table S6). There was an overlap in the top 20 genes of three topological features (Table S7). Furthermore, we identified 42 sub-PPI networks. Among them, sub-network 1 and sub-network 2 were highly interconnected modules for their high node score (Figure 3b, C). The two sub-networks were extracted for functional enrichment analysis. The enriched GO terms of sub-network 1 were closely associated with post-translational protein modification, endoplasmic reticulum lumen, and receptor−ligand activity (Figure S3A), while sub-network 2 was related to extracellular structure organization, ECM organization, and collagen-containing ECM (Figure S3B). In KEGG pathway analysis, sub-network 1 was involved in the chemokine signaling pathway, cytokine−cytokine receptor interaction, and viral protein interaction with cytokines and cytokine receptors (Figure S3C), while sub-network 2 was involved in protein digestion and absorption, the AGE−RAGE signaling pathway in diabetic complications, and human papillomavirus infection (Figure S3D).

### CeRNA network

**Figure 4.**
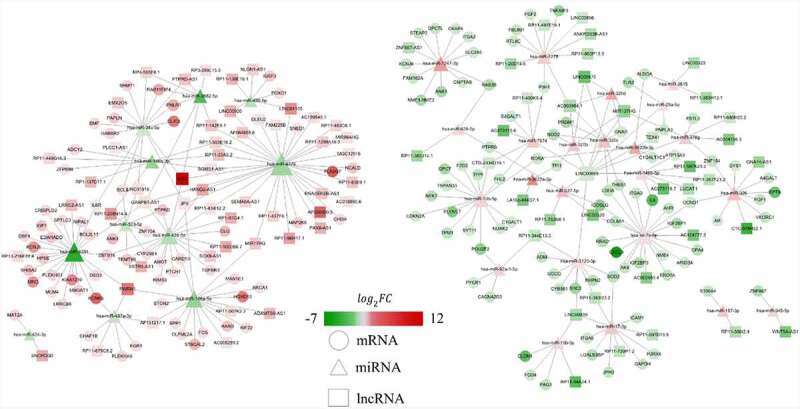
Co-expression network of differentially expressed lncRNAs, miRNAs and mRNAs. Red represents up-regulated expression, whereas green represents down-regulated expression. Circular nodes represent mRNAs, triangular nodes represent miRNAs and square nodes represent lncRNAs. FC, fold change; lncRNAs, long noncoding RNAs; miRNAs, microRNAs

**Figure 5.**
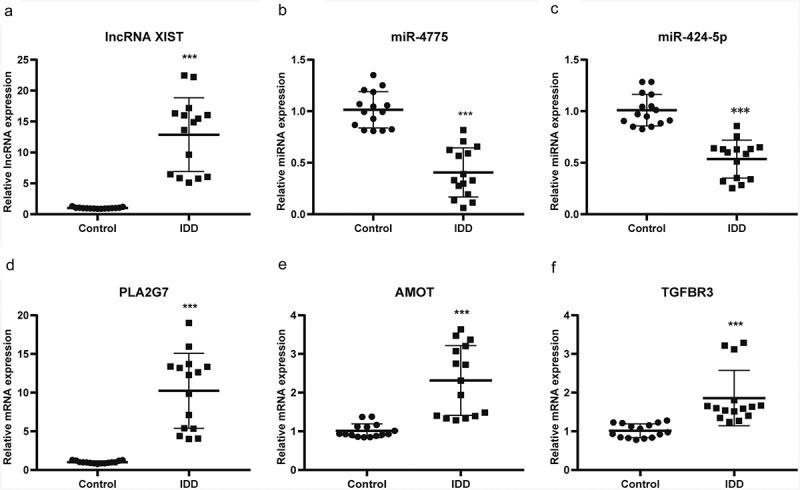
The relative expression level of differentially-expressed genes in ceRNA axes. (a) The relative expression level of lncRNA XIST. (b) The relative expression level of miR-4775. (c) The relative expression level of miR-424-5p. (d) The relative expression level of PLA2G7. (e) The relative expression level of AMOT. (f) The relative expression level of TGFBR3. Mean ± SD, n = 15; The two-tailed Student t-test was used to assess statistical significance: ***P < 0.001. CeRNA, competing endogenous RNA; lncRNA, long noncoding RNA

Furthermore, a lncRNA*–*miRNA*–*mRNA ceRNA network was established to identify potential biomarkers among DELs, DEMis and DEMs. As presented in Figure 4, the ceRNA regulatory network included 90 lncRNA nodes, 34 miRNA nodes, 147 mRNA nodes and 313 edges, which create two particularly important ceRNA axes, including lncRNA XIST (lncRNA ENSG00000229807)–hsa-miR-4775–PLA2G7 and lncRNA XIST–hsa-miR-424-5p–AMOT/TGFBR3. Further, the differentially-expressed level of lncRNA XIST, hsa-miR-4775, hsa-miR-424-5p, PLA2G7, AMOT and TGFBR3 was validated by qRT-PCR. The expressed level of lncRNA XIST increased 12.8-fold while PLA2G7, AMOT and TGFBR3 increased 10.12-fold, 2.28-fold and 1.83-fold in IDD, respectively (Figure 5a, D, E, F). However, the expressed level of hsa-miR-4775 and hsa-miR-424-5p decreased 2.5-fold and 1.89-fold in IDD, respectively (Figure 5b, C). The results showed a good correlation between RNA-seq and qRT-PCR. From the results, we speculated that lncRNA XIST may upregulate PLA2G7 by sponging hsa-miR-4775 as well as AMOT and TGFBR3 by sponging hsa-miR-424-5p.

**Figure 6.**
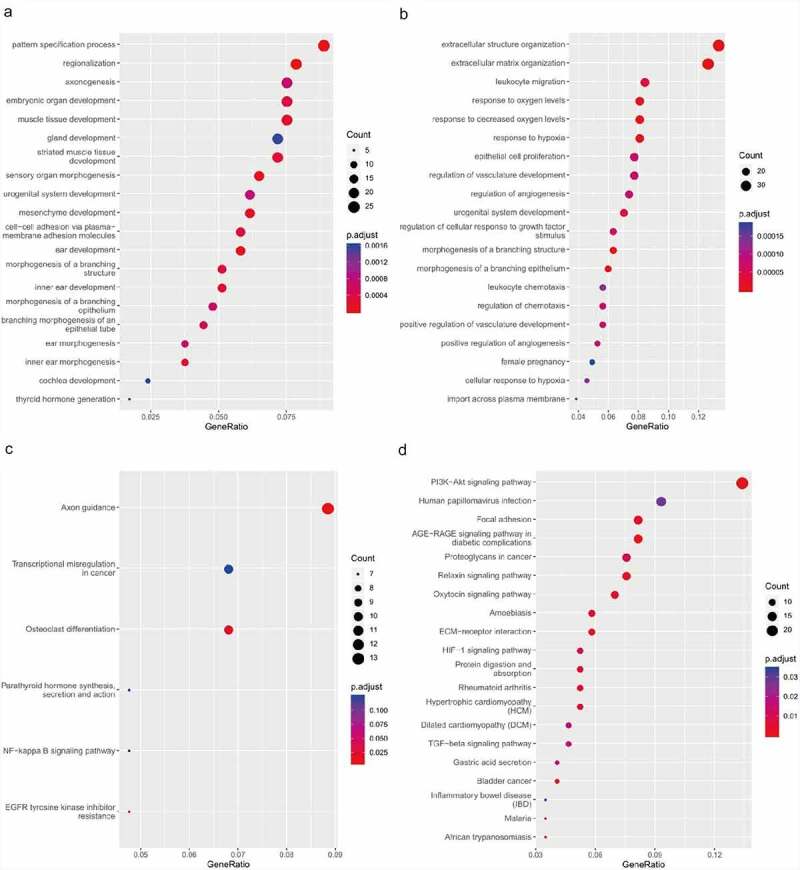
Functional enrichment analysis of co-differentially expressed target genes in ceRNA network based on GO and KEGG. (a) GO analysis showing biological processes of the up-regulated target genes in ceRNA network. (b) GO analysis showing biological processes of the down-regulated target genes in ceRNA network. (c) KEGG pathway analysis of up-regulated target genes in ceRNA network. (d) KEGG pathway analysis of down-regulated target genes in ceRNA network.CeRNA, competing endogenous RNA; GO, gene ontology; KEGG, Kyoto Encyclopedia of Genes and Genomes

To understand the function of DEGs in the ceRNA network, GO and KEGG enrichment analyses were implemented. In the enriched GO terms (biological process) presented in Figure 6a and B, the up-regulated target genes were connected with the pattern specification process, regionalization, and mesenchyme development, whereas the down-regulated target genes were closely connected with ECM organization, extracellular structure organization, and leukocyte migration. The KEGG pathway analyses of up-regulated target genes were connected with axon guidance, osteoclast differentiation, and EGFR tyrosine kinase inhibitor resistance, while the KEGG pathway analysis of the down-regulated target genes was connected with the AGE–RAGE signaling pathway, PI3K–Akt signaling pathway, and relaxin signaling pathway (Figure 6c, D).

## Discussion

In the current study, DELs, DEMis and DEMs in IDD between an IDD group and a control group were identified by RNA-seq. A ceRNA network was constructed and lncRNA XIST–hsa-miR-4775–PLA2G7 and lncRNA XIST–hsa-miR-424-5p–AMOT/TGFBR3 were identified and further validated by qRT-PCR. The outcomes demonstrated a good consistency between RNA-seq and qRT-PCR in this study. The results showed lncRNA XIST was co-expressed with PLA2G7, AMOT and TGFBR3 by regulating hsa-miR-4775 and hsa-miR-424-5p, which revealed that these molecules may act as vital roles during the development of IDD.

In previous studies, Zhu et al. [11] have investigated the interaction mechanisms of lncRNA*–*miRNA*–*mRNA in IDD using public microarray data. However, microarrays are heavily dependent on designed probes and fail to comprehensively explore dynamic and minimally-expressed noncoding RNAs in IDD [14]. Also, noncoding RNAs have distinct tissue specificity and plenty of them have yet to be identified. The RNA-Seq approach is therefore greatly helpful in identifying novel noncoding RNAs [20]. Recently, Zhao et al. [14] identified DELs and DEMs in IDD by RNA-seq and revealed some vital DELs and DEMs in IDD. They further built a ceRNA network with DEMis identified from previous microarray analysis. However, the DEGs were obtained by different detection approaches (RNA-seq and microarray), which may produce biased results. In the present study, we randomly selected three samples each from an IDD and a spinal cord injury group for RNA-seq. Furthermore, the expression profiles of lncRNAs, miRNAs and mRNAs were studied by bioinformatics analysis. The lncRNA–miRNA–mRNA ceRNA network was constructed and some novel and important DEGs were validated in the ceRNA network by qRT-PCR.

In the ceRNA network, two important axes were identified: lncRNA XIST–hsa-miR-4775–PLA2G7 and lncRNA XIST–hsa-miR-424-5p–AMOT/TGFBR3 ceRNA axes. It is worth noting that the lncRNA XIST was significantly differentially-expressed and co-expressed with PLA2G7, AMOT and TGFBR3. As a pro-inflammatory agent, lncRNA XIST has been confirmed to act in neuropathic pain and osteoarthritic synovium [21,22]. However, the biological role of lncRNA XIST in IDD has not been studied. Since inflammation plays an important role during the development of IDD [3], lncRNA XIST may be a crucial molecule in the pathological process of IDD. Besides, PLA2G7 has been confirmed to be involved in inflammation [23]. Further study showed that PLA2G is an important gene that contributes to the pathological process in IDD, and is up-regulated in patients with IDD [24]. In addition, AMOT promotes angiogenesis which is one of the important characteristics of IDD [25,26]. It indicates that AMOT may be involved in the pathological process of IDD. Interestingly, TGFBR3 was up-regulated in IDD, which may be an adaptation to degenerative changes in the disc. Because TGFB3 can promote cell proliferation and maintain ECM [27], as a protective gene, TGFBR3 may be adaptively upregulated during the development of IDD.

A previous study showed that Bcl-2 protected normal intervertebral disc tissues from apoptosis [28]. Gruber et al. found that Bcl-2 was significantly down-regulated in animal models of degenerative intervertebral discs [29]. In this study, it was noticed that expression of Bcl-2 was up-regulated in IDD. One possible explanation is that thoracolumbar fractures may induce early caspase-mediated apoptosis of NP cells and down-regulate the anti-apoptotic protein, Bcl-2 [30].

In this study, GO and KEGG enrichment analyses were conducted to explore the function of noncoding RNAs in the ceRNA network. It was revealed that the most important biological processes of the down-regulated target genes were associated with the ECM which is involved in the pathological processes of IDD. As is well known, loss or imbalance of ECM components promotes IDD [31,32]. Among the most enriched KEGG terms of up-regulated target genes based on the ceRNA regulation network, axon guidance, osteoclast differentiation, and EGFR tyrosine kinase inhibitor resistance were associated with annulus fibrosus stem cell differentiation which is involved in the process of IDD [33]. However, among the predicted pathways of down-regulated target genes, the PI3K–Akt pathway was particularly relevant to IDD. It has been shown that activation of the PI3K–Akt pathway alleviates IDD [34,35].

Certain limitations exist in this study. Firstly, the sample size for RNA-Seq was insufficient in both the control and the IDD groups. Increased sample size should be included for RNA-Seq and qRT-PCR in further studies. Secondly, non-degenerative specimens from spinal cord injury were used as control samples. The results may be affected by local inflammation as well as related post-trauma reactions. In addition, RNA-Seq analysis may be confused by the different genetic backgrounds between the IDD group and controls. Finally, the results of RNA-Seq analysis and qRT-PCR only provide a preliminary screening study result. It will be necessary to carry out multiple experimental studies to validate the regulatory mechanisms of lncRNA*–*miRNA*–*mRNA in IDD.

## Conclusion

In this study, we identified 463 DELs including 279 up-regulated DELs and 184 down-regulated DELs in the IDD group. We also detected 47 DEMis including 29 up-regulated DEMis and 18 down-regulated DEMis. Among the DELs, 358 lncRNAs, acting as cis-regulators, could potentially target 865 genes. PPI network analysis confirmed that IL-6, VEGFA, IGF1, MMP9, CXCL8, FGF2, IL-1B, CCND1, ITGAM, PTPRC, FOS and PTGS2 were hub genes. Furthermore, we constructed a ceRNA regulatory network including 90 lncRNA nodes, 34 miRNA nodes, 147 mRNA nodes and 313 edges. From the ceRNA regulatory network, two ceRNA axes, lncRNA XIST – hsa-miR-4775–PLA2G7 and lncRNA XIST – hsa-miR-424-5p–AMOT/TGFBR3, were identified and further validated by qRT-PCR. The biological processes and signaling pathways of DEGs were predicted by bioinformatics analysis, which may help understand the pathological process of IDD. This study revealed that some specific noncoding RNAs and ceRNA axes were vital during the development of IDD, and may provide available candidate diagnostic biomarkers and novel therapeutic strategies for IDD.

## Supplementary Material

Supplemental Material

## Highlights

1. A total of 463 DELs, 47 DEMis and 1,334 DEMs were identified by RNA-seq in IDD.

2. LncRNA XIST, PLA2G7 and AMOT were risk factors in IDD.

3. Over-expressed lncRNA XIST upregulated PLA2G7 by sponging hsa-miR-4775 in IDD.

4. Over-expressed lncRNA XIST upregulated AMOT by sponging hsa-miR-424-5p in IDD.

5. LncRNA XIST may play a vital role in the process of IDD.

## Data accessibility

Data and analyses can be obtained from the corresponding author on a reasonable request. Sequence data of this study has been uploaded to NCBI and GEO accession number is GSE167199.
