# Supplementary material for: Differentially-expressed mRNAs, microRNAs and long noncoding RNAs in intervertebral disc degeneration identified by RNA-sequencing
Source: Bioengineered. 2021 Mar 25;12(1):1026–39. doi: 10.1080/21655979.2021.1899533 (PMC8806213; doi:10.1080/21655979.2021.1899533)
Supplement: Supplemental Material [file KBIE_A_1899533_SM6208.docx]

**Table S1.** Basic information of the specimens.

| Sample | Age | Gender | MRI grade |  |  |
| --- | --- | --- | --- | --- | --- |
| Control 1 | 35 | M | II |  |  |
| Control 2 | 31 | F | I |  |  |
| Control 3 | 29 | M | I |  |  |
| Control 4 | 32 | M | I |  |  |
| Control 5 | 36 | F | II |  |  |
| Control 6 | 34 | M | I |  |  |
| Control 7 | 35 | F | I |  |  |
| Control 8 | 35 | F | I |  |  |
| Control 9 | 33 | M | I |  |  |
| Control 10 | 37 | M | II |  |  |
| Control 11 | 34 | M | II |  |  |
| Control 12 | 36 | F | II |  |  |
| Control 13 | 35 | M | II |  |  |
| Control 14 | 37 | F | II |  |  |
| Control 15 | 38 | M | II |  |  |
| IDD 1 | 57 | F | IV |  |  |
| IDD 2 | 65 | F | V |  |  |
| IDD 3 | 62 | M | V |  |  |
| IDD 4 | 66 | M | IV |  |  |
| IDD 5 | 68 | M | V |  |  |
| IDD 6 | 72 | F | V |  |  |
| IDD 7 | 67 | F | V |  |  |
| IDD 8 | 65 | M | V |  |  |
| IDD 9 | 65 | F | IV |  |  |
| IDD 10 | 60 | M | IV |  |  |
| IDD 11 | 59 | F | V |  |  |
| IDD 12 | 55 | F | IV |  |  |
| IDD 13 | 58 | M | V |  |  |
| IDD 14 | 54 | M | IV |  |  |
| IDD 15 | 55 | M | IV |  |  |

IDD, intervertebral disc degeneration; M, male; F, male; MRI, magnetic resonance imaging.

**Table S2.** The top ten upregulated and downregulated lncRNAs between the IDD group and controls.

| Upregulated lncRNAs | | Downregulated lncRNAs | |
| --- | --- | --- | --- |
| Gene ID | Log2FC | Gene ID | Log2FC |
| TCONS_00228970 | 12.79 | TCONS_00132082 | −10.45 |
| ENSG00000229807 | 12.04 | ENSG00000251320 | −6.48 |
| TCONS_00228961 | 11.16 | ENSG00000285768 | −5.70 |
| ENSG00000273295 | 7.26 | ENSG00000287275 | −5.65 |
| ENSG00000226263 | 6.82 | ENSG00000203565 | −5.39 |
| ENSG00000253671 | 6.25 | ENSG00000254887 | −5.32 |
| ENSG00000259905 | 6.23 | ENSG00000253891 | −5.16 |
| ENSG00000231873 | 5.57 | ENSG00000253802 | −5.10 |
| ENSG00000276412 | 5.52 | ENSG00000227744 | −5.10 |
| ENSG00000283982 | 5.50 | ENSG00000222031 | −4.74 |

lncRNAs, long noncoding RNAs; IDD, intervertebral disc degeneration; FC, fold change.

**Table S3.** The top ten upregulated and downregulated miRNAs between the IDD group and controls.

| Upregulated miRNAs | | Downregulated miRNAs | |
| --- | --- | --- | --- |
| Gene ID | Log2FC | Gene ID | Log2FC |
| hsa-miR-3622a-5p | 5.19 | hsa-miR-4284 | −5.78 |
| hsa-miR-10400-5p | 5.12 | hsa-miR-2682-5p | −4.56 |
| hsa-miR-1247-5p | 4.67 | hsa-miR-4485-3p | −3.74 |
| hsa-miR-1247-3p | 4.41 | hsa-miR-137-3p | −3.11 |
| hsa-miR-320d | 3.61 | hsa-miR-146a-5p | −2.77 |
| hsa-miR-3622b-3p | 3.61 | hsa-miR-146a-3p | −2.73 |
| hsa-miR-887-3p | 3.12 | hsa-miR-4775 | −2.56 |
| hsa-miR-627-5p | 2.74 | hsa-miR-3117-3p | −2.05 |
| hsa-miR-326 | 2.67 | hsa-miR-450a-5p | −1.88 |
| hsa-miR-320c | 2.57 | hsa-miR-424-5p | −1.86 |

miRNAs, microRNAs; IDD, intervertebral disc degeneration; FC, fold change.

**Table S4.** The top ten upregulated and downregulated mRNAs between the IDD group and controls.

| Upregulated mRNAs | | Downregulated mRNAs | |
| --- | --- | --- | --- |
| Gene ID | Log2FC | Gene ID | Log2FC |
| COL9A3 | 9.55 | MMP1 | −8.33 |
| MMP9 | 9.03 | IL36B | −8.28 |
| LYZ | 8.74 | CXCL6 | −7.30 |
| CD84 | 7.80 | STC1 | −7.18 |
| PLA2G7 | 7.67 | BEX1 | −7.07 |
| AC004556.3 | 7.06 | CXCL10 | −6.82 |
| KCNK5 | 6.98 | PSG4 | −6.77 |
| SMN2 | 6.81 | CXCL8 | −6.69 |
| LILRB4 | 6.70 | OR7D2 | −6.48 |
| ITIH6 | 6.66 | CXCL5 | −6.40 |

IDD, intervertebral disc degeneration; FC, fold change.

**Table S5.** Prediction of potential target genes of lncRNAs.

| lncRNA ID | lncRNA gene name | Chrom | lncRNA start | lncRNA end | Target gene | lncRNA target symbol |
| --- | --- | --- | --- | --- | --- | --- |
| TCONS_00196934 | NA | 7 | 45940462 | 45993970 | ENSG00000283247 | CCDC201 |
| TCONS_00196934 | NA | 7 | 45940462 | 45993970 | ENSG00000146674 | IGFBP3 |
| TCONS_00196934 | NA | 7 | 45940462 | 45993970 | ENSG00000146678 | IGFBP1 |
| ENSG00000225649 | AC064875.1 | 2 | 12780593 | 13007029 | ENSG00000071575 | TRIB2 |
| ENSG00000237471 | AC073115.2 | 7 | 45940449 | 45986472 | ENSG00000283247 | CCDC201 |
| ENSG00000237471 | AC073115.2 | 7 | 45940449 | 45986472 | ENSG00000146674 | IGFBP3 |
| ENSG00000237471 | AC073115.2 | 7 | 45940449 | 45986472 | ENSG00000146678 | IGFBP1 |
| ENSG00000189223 | PAX8-AS1 | 2 | 113211421 | 113276581 | ENSG00000125618 | PAX8 |
| ENSG00000189223 | PAX8-AS1 | 2 | 113211421 | 113276581 | ENSG00000125637 | PSD4 |
| ENSG00000189223 | PAX8-AS1 | 2 | 113211421 | 113276581 | ENSG00000136689 | IL1RN |
| TCONS_00132082 | NA | 2 | 113036279 | 113054317 | ENSG00000136694 | IL36A |
| TCONS_00132082 | NA | 2 | 113036279 | 113054317 | ENSG00000136695 | IL36RN |
| TCONS_00132082 | NA | 2 | 113036279 | 113054317 | ENSG00000136697 | IL1F10 |
| TCONS_00132082 | NA | 2 | 113036279 | 113054317 | ENSG00000136688 | IL36G |
| TCONS_00132082 | NA | 2 | 113036279 | 113054317 | ENSG00000136696 | IL36B |
| TCONS_00132082 | NA | 2 | 113036279 | 113054317 | ENSG00000136689 | IL1RN |
| ENSG00000246763 | RGMB-AS1 | 5 | 98769618 | 98773469 | ENSG00000153922 | CHD1 |
| ENSG00000246763 | RGMB-AS1 | 5 | 98769618 | 98773469 | ENSG00000174136 | RGMB |
| TCONS_00181772 | NA | 5 | 98769181 | 98773556 | ENSG00000153922 | CHD1 |
| TCONS_00181772 | NA | 5 | 98769181 | 98773556 | ENSG00000174136 | RGMB |
| TCONS_00217556 | NA | 9 | 90997040 | 91001901 | ENSG00000165025 | SYK |
| ENSG00000248388 | AC093801.1 | 4 | 176669621 | 176706145 | ENSG00000150630 | VEGFC |
| TCONS_00061771 | NA | 14 | 73265964 | 73274867 | ENSG00000080815 | PSEN1 |
| TCONS_00061771 | NA | 14 | 73265964 | 73274867 | ENSG00000100767 | PAPLN |
| TCONS_00061771 | NA | 14 | 73265964 | 73274867 | ENSG00000133961 | NUMB |
| ENSG00000225206 | MIR137HG | 1 | 97933474 | 98049863 | ENSG00000188641 | DPYD |
| ENSG00000260454 | AL355607.2 | 9 | 90996949 | 91001901 | ENSG00000165025 | SYK |
| TCONS_00212776 | NA | 8 | 56515445 | 56559557 | ENSG00000181195 | PENK |
| TCONS_00190100 | NA | 6 | 3722620 | 3752098 | ENSG00000145945 | FAM50B |
| TCONS_00190100 | NA | 6 | 3722620 | 3752098 | ENSG00000168994 | PXDC1 |
| ENSG00000235385 | LINC02154 | X | 13266048 | 13303452 | ENSG00000123594 | ATXN3L |
| ENSG00000234899 | SOX9-AS1 | 17 | 72034107 | 72237203 | ENSG00000125398 | SOX9 |
| ENSG00000248323 | LUCAT1 | 5 | 91054834 | 91314547 | ENSG00000113369 | ARRDC3 |
| ENSG00000248323 | LUCAT1 | 5 | 91054834 | 91314547 | ENSG00000164199 | ADGRV1 |
| ENSG00000260578 | AC110597.1 | 18 | 67481791 | 67484966 | ENSG00000171451 | DSEL |
| ENSG00000255400 | AC124276.2 | 11 | 12086891 | 12089441 | ENSG00000050165 | DKK3 |
| ENSG00000255400 | AC124276.2 | 11 | 12086891 | 12089441 | ENSG00000133816 | MICAL2 |
| ENSG00000166770 | ZNF667-AS1 | 19 | 56477250 | 56504362 | ENSG00000196263 | ZNF471 |
| ENSG00000166770 | ZNF667-AS1 | 19 | 56477250 | 56504362 | ENSG00000198046 | ZNF667 |
| ENSG00000166770 | ZNF667-AS1 | 19 | 56477250 | 56504362 | ENSG00000018869 | ZNF582 |
| ENSG00000166770 | ZNF667-AS1 | 19 | 56477250 | 56504362 | ENSG00000196867 | ZFP28 |
| ENSG00000166770 | ZNF667-AS1 | 19 | 56477250 | 56504362 | ENSG00000198440 | ZNF583 |
| ENSG00000166770 | ZNF667-AS1 | 19 | 56477250 | 56504362 | ENSG00000197016 | ZNF470 |
| ENSG00000166770 | ZNF667-AS1 | 19 | 56477250 | 56504362 | ENSG00000197951 | ZNF71 |
| ENSG00000185904 | LINC00839 | 10 | 42475480 | 42495337 | ENSG00000196693 | ZNF33B |
| ENSG00000250095 | NREP-AS1 | 5 | 111912508 | 112017309 | ENSG00000134986 | NREP |
| TCONS_00123034 | NA | 2 | 113235655 | 113273056 | ENSG00000125618 | PAX8 |
| TCONS_00123034 | NA | 2 | 113235655 | 113273056 | ENSG00000125637 | PSD4 |
| ENSG00000246430 | LINC00968 | 8 | 56496048 | 56559823 | ENSG00000181195 | PENK |
| ENSG00000237499 | WAKMAR2 | 6 | 137823673 | 137868233 | ENSG00000118503 | TNFAIP3 |
| ENSG00000260910 | LINC00565 | 13 | 113926514 | 113928844 | ENSG00000183087 | GAS6 |
| ENSG00000260910 | LINC00565 | 13 | 113926514 | 113928844 | ENSG00000283199 | C13orf46 |
| ENSG00000260910 | LINC00565 | 13 | 113926514 | 113928844 | ENSG00000185989 | RASA3 |
| ENSG00000229628 | AC073115.1 | 7 | 45990905 | 46000898 | ENSG00000146674 | IGFBP3 |
| ENSG00000229628 | AC073115.1 | 7 | 45990905 | 46000898 | ENSG00000146678 | IGFBP1 |
| ENSG00000230815 | AL807757.1 | 9 | 94824272 | 94824773 | ENSG00000148120 | AOPEP |
| ENSG00000262202 | AC007952.4 | 17 | 19112000 | 19112636 | ENSG00000154016 | GRAP |
| ENSG00000262202 | AC007952.4 | 17 | 19112000 | 19112636 | ENSG00000189152 | GRAPL |
| ENSG00000262202 | AC007952.4 | 17 | 19112000 | 19112636 | ENSG00000154025 | SLC5A10 |
| TCONS_00123012 | NA | 2 | 113235149 | 113242124 | ENSG00000125618 | PAX8 |
| TCONS_00123012 | NA | 2 | 113235149 | 113242124 | ENSG00000125637 | PSD4 |
| TCONS_00123039 | NA | 2 | 113250186 | 113276581 | ENSG00000125618 | PAX8 |
| TCONS_00123039 | NA | 2 | 113250186 | 113276581 | ENSG00000125637 | PSD4 |
| ENSG00000270640 | AC104695.4 | 2 | 28396815 | 28397110 | ENSG00000075426 | FOSL2 |
| ENSG00000270640 | AC104695.4 | 2 | 28396815 | 28397110 | ENSG00000163803 | PLB1 |
| ENSG00000270640 | AC104695.4 | 2 | 28396815 | 28397110 | ENSG00000158019 | BABAM2 |
| ENSG00000215417 | MIR17HG | 13 | 91347820 | 91354579 | ENSG00000179399 | GPC5 |
| ENSG00000273295 | AP000350.6 | 22 | 23901432 | 23907068 | ENSG00000276950 | GSTT4 |
| ENSG00000273295 | AP000350.6 | 22 | 23901432 | 23907068 | ENSG00000240972 | MIF |
| ENSG00000273295 | AP000350.6 | 22 | 23901432 | 23907068 | ENSG00000251357 | AP000350.4 |
| ENSG00000273295 | AP000350.6 | 22 | 23901432 | 23907068 | ENSG00000285762 | AC253536.7 |
| ENSG00000273295 | AP000350.6 | 22 | 23901432 | 23907068 | ENSG00000133433 | GSTT2B |
| ENSG00000273295 | AP000350.6 | 22 | 23901432 | 23907068 | ENSG00000099974 | DDTL |
| ENSG00000273295 | AP000350.6 | 22 | 23901432 | 23907068 | ENSG00000099958 | DERL3 |
| ENSG00000273295 | AP000350.6 | 22 | 23901432 | 23907068 | ENSG00000099956 | SMARCB1 |
| ENSG00000273295 | AP000350.6 | 22 | 23901432 | 23907068 | ENSG00000133460 | SLC2A11 |
| ENSG00000273295 | AP000350.6 | 22 | 23901432 | 23907068 | ENSG00000099977 | DDT |
| ENSG00000280241 | AC079298.3 | 4 | 153948718 | 154300500 | ENSG00000197410 | DCHS2 |
| TCONS_00208920 | NA | 8 | 71956982 | 72062292 | ENSG00000104321 | TRPA1 |
| ENSG00000235531 | MSC-AS1 | 8 | 71828167 | 72118393 | ENSG00000104321 | TRPA1 |
| ENSG00000235531 | MSC-AS1 | 8 | 71828167 | 72118393 | ENSG00000178860 | MSC |
| ENSG00000244586 | WNT5A-AS1 | 3 | 55487699 | 55488308 | ENSG00000114251 | WNT5A |
| ENSG00000244586 | WNT5A-AS1 | 3 | 55487699 | 55488308 | ENSG00000187672 | ERC2 |
| ENSG00000136275 | C7orf69 | 7 | 47795291 | 47819847 | ENSG00000158683 | PKD1L1 |
| ENSG00000240032 | LNCSRLR | 3 | 146066344 | 146069185 | ENSG00000152952 | PLOD2 |
| ENSG00000206195 | DUXAP8 | 22 | 15784959 | 15829984 | ENSG00000198062 | POTEH |
| ENSG00000261051 | AC107021.2 | 3 | 146059585 | 146061679 | ENSG00000152952 | PLOD2 |
| ENSG00000253671 | AC027117.1 | 8 | 17808361 | 17822183 | ENSG00000129422 | MTUS1 |
| ENSG00000253671 | AC027117.1 | 8 | 17808361 | 17822183 | ENSG00000104760 | FGL1 |
| ENSG00000229647 | MYOSLID | 2 | 207166120 | 207248668 | ENSG00000118263 | KLF7 |
| ENSG00000243415 | AC107021.1 | 3 | 146064042 | 146105204 | ENSG00000114698 | PLSCR4 |
| ENSG00000243415 | AC107021.1 | 3 | 146064042 | 146105204 | ENSG00000152952 | PLOD2 |
| TCONS_00041519 | NA | 11 | 122028395 | 122119229 | ENSG00000259571 | BLID |
| TCONS_00187496 | NA | 6 | 63521748 | 63583587 | ENSG00000118482 | PHF3 |
| TCONS_00187496 | NA | 6 | 63521748 | 63583587 | ENSG00000112245 | PTP4A1 |
| TCONS_00187496 | NA | 6 | 63521748 | 63583587 | ENSG00000285976 | AL135905.2 |
| ENSG00000262119 | AL079343.1 | 14 | 26592747 | 26594517 | ENSG00000139910 | NOVA1 |
| ENSG00000245552 | AP000787.1 | 11 | 95150539 | 95234391 | ENSG00000263465 | SRSF8 |
| ENSG00000245552 | AP000787.1 | 11 | 95150539 | 95234391 | ENSG00000149212 | SESN3 |
| ENSG00000245552 | AP000787.1 | 11 | 95150539 | 95234391 | ENSG00000255855 | KDM4F |
| ENSG00000245552 | AP000787.1 | 11 | 95150539 | 95234391 | ENSG00000149218 | ENDOD1 |
| ENSG00000198468 | FLVCR1-DT | 1 | 212852105 | 212858126 | ENSG00000143494 | VASH2 |
| ENSG00000198468 | FLVCR1-DT | 1 | 212852105 | 212858126 | ENSG00000162769 | FLVCR1 |
| ENSG00000198468 | FLVCR1-DT | 1 | 212852105 | 212858126 | ENSG00000185523 | SPATA45 |
| ENSG00000198468 | FLVCR1-DT | 1 | 212852105 | 212858126 | ENSG00000203705 | TATDN3 |
| ENSG00000198468 | FLVCR1-DT | 1 | 212852105 | 212858126 | ENSG00000117697 | NSL1 |
| ENSG00000130600 | H19 | 11 | 1995176 | 2001470 | ENSG00000130595 | TNNT3 |
| ENSG00000130600 | H19 | 11 | 1995176 | 2001470 | ENSG00000214026 | MRPL23 |
| TCONS_00063058 | NA | 14 | 100894699 | 101006021 | ENSG00000254656 | RTL1 |
| ENSG00000253837 | AC090197.1 | 8 | 23336171 | 23366125 | ENSG00000134013 | LOXL2 |
| ENSG00000253837 | AC090197.1 | 8 | 23336171 | 23366125 | ENSG00000147457 | CHMP7 |
| ENSG00000253837 | AC090197.1 | 8 | 23336171 | 23366125 | ENSG00000104679 | R3HCC1 |
| ENSG00000253837 | AC090197.1 | 8 | 23336171 | 23366125 | ENSG00000197217 | ENTPD4 |
| TCONS_00109585 | NA | 19 | 39406857 | 39409412 | ENSG00000105197 | TIMM50 |
| TCONS_00109585 | NA | 19 | 39406857 | 39409412 | ENSG00000006712 | PAF1 |
| TCONS_00109585 | NA | 19 | 39406857 | 39409412 | ENSG00000090932 | DLL3 |
| TCONS_00109585 | NA | 19 | 39406857 | 39409412 | ENSG00000179134 | SAMD4B |
| TCONS_00109585 | NA | 19 | 39406857 | 39409412 | ENSG00000090924 | PLEKHG2 |
| TCONS_00109585 | NA | 19 | 39406857 | 39409412 | ENSG00000105193 | RPS16 |
| TCONS_00109585 | NA | 19 | 39406857 | 39409412 | ENSG00000128011 | LRFN1 |
| TCONS_00109585 | NA | 19 | 39406857 | 39409412 | ENSG00000063322 | MED29 |
| TCONS_00109585 | NA | 19 | 39406857 | 39409412 | ENSG00000128016 | ZFP36 |
| TCONS_00109585 | NA | 19 | 39406857 | 39409412 | ENSG00000130755 | GMFG |
| TCONS_00109585 | NA | 19 | 39406857 | 39409412 | ENSG00000196235 | SUPT5H |
| TCONS_00018595 | NA | 1 | 200412627 | 200483604 | ENSG00000118193 | KIF14 |
| TCONS_00018595 | NA | 1 | 200412627 | 200483604 | ENSG00000162702 | ZNF281 |
| TCONS_00157724 | NA | 3 | 27443293 | 27484420 | ENSG00000033867 | SLC4A7 |
| TCONS_00157724 | NA | 3 | 27443293 | 27484420 | ENSG00000163491 | NEK10 |
| ENSG00000228065 | LINC01515 | 10 | 65570338 | 65880289 | ENSG00000183230 | CTNNA3 |
| ENSG00000244879 | GABPB1-AS1 | 15 | 50354959 | 50372202 | ENSG00000138592 | USP8 |
| ENSG00000244879 | GABPB1-AS1 | 15 | 50354959 | 50372202 | ENSG00000140287 | HDC |
| ENSG00000244879 | GABPB1-AS1 | 15 | 50354959 | 50372202 | ENSG00000104064 | GABPB1 |
| ENSG00000256650 | RERG-IT1 | 12 | 15112363 | 15114698 | ENSG00000134533 | RERG |
| ENSG00000226686 | LINC01535 | 19 | 37251885 | 37265535 | ENSG00000188283 | ZNF383 |
| ENSG00000226686 | LINC01535 | 19 | 37251885 | 37265535 | ENSG00000196967 | ZNF585A |
| ENSG00000226686 | LINC01535 | 19 | 37251885 | 37265535 | ENSG00000267360 | AC012309.1 |
| ENSG00000226686 | LINC01535 | 19 | 37251885 | 37265535 | ENSG00000181666 | ZNF875 |
| ENSG00000226686 | LINC01535 | 19 | 37251885 | 37265535 | ENSG00000245680 | ZNF585B |
| ENSG00000259518 | LINC01583 | 15 | 82088569 | 82097694 | ENSG00000140598 | EFL1 |
| ENSG00000259518 | LINC01583 | 15 | 82088569 | 82097694 | ENSG00000183496 | MEX3B |
| ENSG00000261618 | LINC02605 | 8 | 78835307 | 78840525 | ENSG00000104432 | IL7 |
| TCONS_00217207 | NA | 9 | 76700180 | 76752115 | ENSG00000106772 | PRUNE2 |
| TCONS_00217207 | NA | 9 | 76700180 | 76752115 | ENSG00000187210 | GCNT1 |
| TCONS_00076267 | NA | 15 | 100912100 | 100919391 | ENSG00000184254 | ALDH1A3 |
| TCONS_00076267 | NA | 15 | 100912100 | 100919391 | ENSG00000154237 | LRRK1 |
| ENSG00000226263 | ISM1-AS1 | 20 | 13237801 | 13239674 | ENSG00000172296 | SPTLC3 |
| ENSG00000226263 | ISM1-AS1 | 20 | 13237801 | 13239674 | ENSG00000101230 | ISM1 |
| ENSG00000236049 | LINC01920 | 2 | 150552532 | 150572221 | ENSG00000115963 | RND3 |
| ENSG00000261340 | LINC01616 | 11 | 29980113 | 29982392 | ENSG00000182255 | KCNA4 |
| ENSG00000261335 | AC005837.1 | 17 | 76671942 | 76673658 | ENSG00000070526 | ST6GALNAC1 |
| ENSG00000261335 | AC005837.1 | 17 | 76671942 | 76673658 | ENSG00000161547 | SRSF2 |
| ENSG00000261335 | AC005837.1 | 17 | 76671942 | 76673658 | ENSG00000182534 | MXRA7 |
| ENSG00000261335 | AC005837.1 | 17 | 76671942 | 76673658 | ENSG00000070731 | ST6GALNAC2 |
| ENSG00000261335 | AC005837.1 | 17 | 76671942 | 76673658 | ENSG00000070495 | JMJD6 |
| ENSG00000261335 | AC005837.1 | 17 | 76671942 | 76673658 | ENSG00000181038 | METTL23 |
| ENSG00000261335 | AC005837.1 | 17 | 76671942 | 76673658 | ENSG00000267168 | AC005837.2 |
| ENSG00000261335 | AC005837.1 | 17 | 76671942 | 76673658 | ENSG00000092931 | MFSD11 |
| TCONS_00209774 | NA | 8 | 106270218 | 106329010 | ENSG00000164830 | OXR1 |
| ENSG00000225937 | PCA3 | 9 | 76691980 | 76863307 | ENSG00000106772 | PRUNE2 |
| ENSG00000225937 | PCA3 | 9 | 76691980 | 76863307 | ENSG00000187210 | GCNT1 |
| ENSG00000260876 | LINC01229 | 16 | 79676048 | 79809716 | ENSG00000178573 | MAF |
| ENSG00000224048 | LINC02612 | 2 | 150612381 | 150635818 | ENSG00000115963 | RND3 |
| TCONS_00065194 | NA | 14 | 49853616 | 49862914 | ENSG00000165516 | KLHDC2 |
| TCONS_00065194 | NA | 14 | 49853616 | 49862914 | ENSG00000165525 | NEMF |
| TCONS_00065194 | NA | 14 | 49853616 | 49862914 | ENSG00000165527 | ARF6 |
| ENSG00000215068 | AC025171.2 | 5 | 43041575 | 43054603 | ENSG00000172262 | ZNF131 |
| ENSG00000215068 | AC025171.2 | 5 | 43041575 | 43054603 | ENSG00000177721 | ANXA2R |
| TCONS_00206030 | NA | 7 | 151402103 | 151410038 | ENSG00000127377 | CRYGN |
| TCONS_00206030 | NA | 7 | 151402103 | 151410038 | ENSG00000187260 | WDR86 |
| TCONS_00206030 | NA | 7 | 151402103 | 151410038 | ENSG00000106615 | RHEB |
| TCONS_00206030 | NA | 7 | 151402103 | 151410038 | ENSG00000013374 | NUB1 |
| ENSG00000231607 | DLEU2 | 13 | 49956670 | 50125720 | ENSG00000123178 | SPRYD7 |
| ENSG00000231607 | DLEU2 | 13 | 49956670 | 50125720 | ENSG00000204977 | TRIM13 |
| ENSG00000231607 | DLEU2 | 13 | 49956670 | 50125720 | ENSG00000198553 | KCNRG |
| TCONS_00099390 | NA | 17 | 72071042 | 72119543 | ENSG00000125398 | SOX9 |
| ENSG00000272695 | GAS6-DT | 13 | 113864112 | 113866834 | ENSG00000184497 | TMEM255B |
| ENSG00000272695 | GAS6-DT | 13 | 113864112 | 113866834 | ENSG00000183087 | GAS6 |
| ENSG00000272695 | GAS6-DT | 13 | 113864112 | 113866834 | ENSG00000283199 | C13orf46 |
| ENSG00000231246 | AL445426.1 | 1 | 112177234 | 112360625 | ENSG00000143079 | CTTNBP2NL |
| ENSG00000281344 | HELLPAR | 12 | 102197585 | 102402596 | ENSG00000183395 | PMCH |
| ENSG00000281344 | HELLPAR | 12 | 102197585 | 102402596 | ENSG00000017427 | IGF1 |
| ENSG00000281344 | HELLPAR | 12 | 102197585 | 102402596 | ENSG00000185480 | PARPBP |
| ENSG00000281344 | HELLPAR | 12 | 102197585 | 102402596 | ENSG00000075188 | NUP37 |
| ENSG00000231160 | KLF3-AS1 | 4 | 38602438 | 38664914 | ENSG00000109787 | KLF3 |
| ENSG00000287872 | AC026956.2 | 15 | 82113384 | 82119570 | ENSG00000140598 | EFL1 |
| ENSG00000287872 | AC026956.2 | 15 | 82113384 | 82119570 | ENSG00000183496 | MEX3B |
| ENSG00000282849 | AL359834.1 | 1 | 200478020 | 200483604 | ENSG00000118193 | KIF14 |
| ENSG00000282849 | AL359834.1 | 1 | 200478020 | 200483604 | ENSG00000162702 | ZNF281 |
| TCONS_00124938 | NA | 2 | 178413970 | 178440243 | ENSG00000180228 | PRKRA |
| TCONS_00124938 | NA | 2 | 178413970 | 178440243 | ENSG00000079150 | FKBP7 |
| TCONS_00124938 | NA | 2 | 178413970 | 178440243 | ENSG00000079156 | OSBPL6 |
| TCONS_00124938 | NA | 2 | 178413970 | 178440243 | ENSG00000116095 | PLEKHA3 |
| TCONS_00124938 | NA | 2 | 178413970 | 178440243 | ENSG00000155657 | TTN |
| TCONS_00124938 | NA | 2 | 178413970 | 178440243 | ENSG00000204311 | PJVK |
| ENSG00000205562 | AL049775.1 | 14 | 85518871 | 85531585 | ENSG00000185070 | FLRT2 |
| ENSG00000254710 | AP001970.1 | 11 | 123188802 | 123228502 | ENSG00000166250 | CLMP |
| ENSG00000251320 | AC011352.3 | 5 | 147887112 | 147887704 | ENSG00000178776 | C5orf46 |
| ENSG00000251320 | AC011352.3 | 5 | 147887112 | 147887704 | ENSG00000164265 | SCGB3A2 |
| ENSG00000251320 | AC011352.3 | 5 | 147887112 | 147887704 | ENSG00000164266 | SPINK1 |
| ENSG00000261327 | AC134312.5 | 16 | 88177298 | 88186929 | ENSG00000172530 | BANP |
| ENSG00000249252 | AC098829.1 | 4 | 15004942 | 15427914 | ENSG00000137449 | CPEB2 |
| ENSG00000249252 | AC098829.1 | 4 | 15004942 | 15427914 | ENSG00000048342 | CC2D2A |
| ENSG00000249252 | AC098829.1 | 4 | 15004942 | 15427914 | ENSG00000163145 | C1QTNF7 |
| ENSG00000224945 | AL353150.1 | 9 | 89088604 | 89109934 | ENSG00000148082 | SHC3 |
| ENSG00000224945 | AL353150.1 | 9 | 89088604 | 89109934 | ENSG00000213694 | S1PR3 |
| TCONS_00212734 | NA | 8 | 56067295 | 56069522 | ENSG00000254087 | LYN |
| TCONS_00212734 | NA | 8 | 56067295 | 56069522 | ENSG00000181690 | PLAG1 |
| TCONS_00212734 | NA | 8 | 56067295 | 56069522 | ENSG00000008988 | RPS20 |
| TCONS_00212734 | NA | 8 | 56067295 | 56069522 | ENSG00000172680 | MOS |
| TCONS_00044655 | NA | 12 | 52249300 | 52252316 | ENSG00000167767 | KRT80 |
| TCONS_00044655 | NA | 12 | 52249300 | 52252316 | ENSG00000170442 | KRT86 |
| TCONS_00044655 | NA | 12 | 52249300 | 52252316 | ENSG00000135480 | KRT7 |
| TCONS_00044655 | NA | 12 | 52249300 | 52252316 | ENSG00000170523 | KRT83 |
| TCONS_00044655 | NA | 12 | 52249300 | 52252316 | ENSG00000205426 | KRT81 |
| ENSG00000259905 | PWRN1 | 15 | 24101827 | 24823365 | ENSG00000185823 | NPAP1 |
| ENSG00000259905 | PWRN1 | 15 | 24101827 | 24823365 | ENSG00000128739 | SNRPN |
| TCONS_00200817 | NA | 7 | 151501878 | 151511774 | ENSG00000127377 | CRYGN |
| TCONS_00200817 | NA | 7 | 151501878 | 151511774 | ENSG00000187260 | WDR86 |
| TCONS_00200817 | NA | 7 | 151501878 | 151511774 | ENSG00000106615 | RHEB |
| TCONS_00200817 | NA | 7 | 151501878 | 151511774 | ENSG00000106617 | PRKAG2 |
| ENSG00000246859 | STARD4-AS1 | 5 | 111510396 | 111739726 | ENSG00000164211 | STARD4 |
| ENSG00000246859 | STARD4-AS1 | 5 | 111510396 | 111739726 | ENSG00000134986 | NREP |
| ENSG00000246859 | STARD4-AS1 | 5 | 111510396 | 111739726 | ENSG00000152495 | CAMK4 |
| ENSG00000285973 | AC063960.2 | 10 | 122145106 | 122155197 | ENSG00000138162 | TACC2 |
| TCONS_00015387 | NA | 1 | 109062444 | 109063883 | ENSG00000121940 | CLCC1 |
| TCONS_00015387 | NA | 1 | 109062444 | 109063883 | ENSG00000197780 | TAF13 |
| TCONS_00015387 | NA | 1 | 109062444 | 109063883 | ENSG00000116299 | KIAA1324 |
| TCONS_00015387 | NA | 1 | 109062444 | 109063883 | ENSG00000215717 | TMEM167B |
| TCONS_00015387 | NA | 1 | 109062444 | 109063883 | ENSG00000085433 | WDR47 |
| TCONS_00015387 | NA | 1 | 109062444 | 109063883 | ENSG00000179902 | C1orf194 |
| TCONS_00015387 | NA | 1 | 109062444 | 109063883 | ENSG00000162641 | AKNAD1 |
| ENSG00000262728 | AC123768.3 | 15 | 32585524 | 32615158 | ENSG00000232653 | GOLGA8N |
| ENSG00000262728 | AC123768.3 | 15 | 32585524 | 32615158 | ENSG00000166922 | SCG5 |
| ENSG00000262728 | AC123768.3 | 15 | 32585524 | 32615158 | ENSG00000198826 | ARHGAP11A |
| TCONS_00124831 | NA | 2 | 174663505 | 174666043 | ENSG00000138435 | CHRNA1 |
| TCONS_00124831 | NA | 2 | 174663505 | 174666043 | ENSG00000115935 | WIPF1 |
| TCONS_00049866 | NA | 12 | 24210054 | 24562667 | ENSG00000134532 | SOX5 |
| ENSG00000268812 | AC004264.1 | 22 | 30246205 | 30246998 | ENSG00000099992 | TBC1D10A |
| ENSG00000268812 | AC004264.1 | 22 | 30246205 | 30246998 | ENSG00000176635 | HORMAD2 |
| ENSG00000268812 | AC004264.1 | 22 | 30246205 | 30246998 | ENSG00000128342 | LIF |
| ENSG00000268812 | AC004264.1 | 22 | 30246205 | 30246998 | ENSG00000099985 | OSM |
| ENSG00000268812 | AC004264.1 | 22 | 30246205 | 30246998 | ENSG00000099995 | SF3A1 |
| ENSG00000268812 | AC004264.1 | 22 | 30246205 | 30246998 | ENSG00000248751 | AC004997.1 |
| ENSG00000268812 | AC004264.1 | 22 | 30246205 | 30246998 | ENSG00000239282 | CASTOR1 |
| TCONS_00055765 | NA | 13 | 49999731 | 50004935 | ENSG00000123178 | SPRYD7 |
| TCONS_00055765 | NA | 13 | 49999731 | 50004935 | ENSG00000204977 | TRIM13 |
| TCONS_00055765 | NA | 13 | 49999731 | 50004935 | ENSG00000198553 | KCNRG |
| ENSG00000246214 | AC024588.1 | 5 | 16615926 | 16681905 | ENSG00000154153 | RETREG1 |
| ENSG00000246214 | AC024588.1 | 5 | 16615926 | 16681905 | ENSG00000145555 | MYO10 |
| ENSG00000227744 | LINC01940 | 2 | 238919302 | 238926269 | ENSG00000233608 | TWIST2 |
| ENSG00000203875 | SNHG5 | 6 | 85650491 | 85678932 | ENSG00000271793 | AL589666.1 |
| ENSG00000203875 | SNHG5 | 6 | 85650491 | 85678932 | ENSG00000135317 | SNX14 |
| ENSG00000203875 | SNHG5 | 6 | 85650491 | 85678932 | ENSG00000135316 | SYNCRIP |
| ENSG00000223485 | LINC01615 | 6 | 169157162 | 169162992 | ENSG00000186340 | THBS2 |
| ENSG00000179136 | LINC00670 | 17 | 12549764 | 12642854 | ENSG00000141052 | MYOCD |
| ENSG00000233844 | KCNQ5-IT1 | 6 | 72630495 | 72678558 | ENSG00000185760 | KCNQ5 |
| ENSG00000270084 | GAS5-AS1 | 1 | 173862473 | 173863941 | ENSG00000120334 | CENPL |
| ENSG00000270084 | GAS5-AS1 | 1 | 173862473 | 173863941 | ENSG00000117601 | SERPINC1 |
| ENSG00000270084 | GAS5-AS1 | 1 | 173862473 | 173863941 | ENSG00000185278 | ZBTB37 |
| ENSG00000270084 | GAS5-AS1 | 1 | 173862473 | 173863941 | ENSG00000117593 | DARS2 |
| ENSG00000270084 | GAS5-AS1 | 1 | 173862473 | 173863941 | ENSG00000135870 | RC3H1 |
| ENSG00000270084 | GAS5-AS1 | 1 | 173862473 | 173863941 | ENSG00000076321 | KLHL20 |
| TCONS_00007928 | NA | 1 | 173861307 | 173863941 | ENSG00000120334 | CENPL |
| TCONS_00007928 | NA | 1 | 173861307 | 173863941 | ENSG00000117601 | SERPINC1 |
| TCONS_00007928 | NA | 1 | 173861307 | 173863941 | ENSG00000185278 | ZBTB37 |
| TCONS_00007928 | NA | 1 | 173861307 | 173863941 | ENSG00000117593 | DARS2 |
| TCONS_00007928 | NA | 1 | 173861307 | 173863941 | ENSG00000135870 | RC3H1 |
| TCONS_00007928 | NA | 1 | 173861307 | 173863941 | ENSG00000076321 | KLHL20 |
| ENSG00000287275 | AC011451.4 | 19 | 9458119 | 9462388 | ENSG00000197961 | ZNF121 |
| ENSG00000287275 | AC011451.4 | 19 | 9458119 | 9462388 | ENSG00000270011 | ZNF559-ZNF177 |
| ENSG00000287275 | AC011451.4 | 19 | 9458119 | 9462388 | ENSG00000198028 | ZNF560 |
| ENSG00000287275 | AC011451.4 | 19 | 9458119 | 9462388 | ENSG00000174652 | ZNF266 |
| ENSG00000287275 | AC011451.4 | 19 | 9458119 | 9462388 | ENSG00000130818 | ZNF426 |
| ENSG00000287275 | AC011451.4 | 19 | 9458119 | 9462388 | ENSG00000188629 | ZNF177 |
| ENSG00000250519 | AP002784.1 | 11 | 94185439 | 94282203 | ENSG00000181333 | HEPHL1 |
| ENSG00000250519 | AP002784.1 | 11 | 94185439 | 94282203 | ENSG00000183560 | IZUMO1R |
| ENSG00000250519 | AP002784.1 | 11 | 94185439 | 94282203 | ENSG00000110218 | PANX1 |
| ENSG00000250519 | AP002784.1 | 11 | 94185439 | 94282203 | ENSG00000123901 | GPR83 |
| TCONS_00087150 | NA | 16 | 88177298 | 88187168 | ENSG00000172530 | BANP |
| ENSG00000237943 | PRKCQ-AS1 | 10 | 6580419 | 6616921 | ENSG00000065675 | PRKCQ |
| ENSG00000205622 | AP001042.1 | 21 | 38812878 | 38956467 | ENSG00000157557 | ETS2 |
| ENSG00000241158 | ADAMTS9-AS1 | 3 | 64561322 | 64592757 | ENSG00000163638 | ADAMTS9 |
| TCONS_00219864 | NA | 9 | 3520083 | 3526004 | ENSG00000080298 | RFX3 |
| ENSG00000215386 | MIR99AHG | 21 | 15928296 | 16645467 | ENSG00000155313 | USP25 |
| ENSG00000259194 | AC020891.1 | 15 | 51367377 | 51369110 | ENSG00000186417 | GLDN |
| ENSG00000259194 | AC020891.1 | 15 | 51367377 | 51369110 | ENSG00000137869 | CYP19A1 |
| ENSG00000259194 | AC020891.1 | 15 | 51367377 | 51369110 | ENSG00000104093 | DMXL2 |
| ENSG00000224517 | HTR2A-AS1 | 13 | 46852143 | 46856299 | ENSG00000102468 | HTR2A |
| ENSG00000224517 | HTR2A-AS1 | 13 | 46852143 | 46856299 | ENSG00000136141 | LRCH1 |
| ENSG00000224517 | HTR2A-AS1 | 13 | 46852143 | 46856299 | ENSG00000139684 | ESD |
| ENSG00000233672 | RNASEH2B-AS1 | 13 | 50862172 | 50910764 | ENSG00000186047 | DLEU7 |
| ENSG00000233672 | RNASEH2B-AS1 | 13 | 50862172 | 50910764 | ENSG00000136104 | RNASEH2B |
| ENSG00000260641 | AC114811.2 | 4 | 98658904 | 98664550 | ENSG00000168785 | TSPAN5 |
| ENSG00000259602 | AC091073.1 | 15 | 48938236 | 48948031 | ENSG00000185634 | SHC4 |
| ENSG00000259602 | AC091073.1 | 15 | 48938236 | 48948031 | ENSG00000255302 | EID1 |
| ENSG00000259602 | AC091073.1 | 15 | 48938236 | 48948031 | ENSG00000138593 | SECISBP2L |
| TCONS_00102684 | NA | 18 | 59697425 | 59698125 | ENSG00000183287 | CCBE1 |
| TCONS_00058216 | NA | 13 | 50044515 | 50082041 | ENSG00000204977 | TRIM13 |
| TCONS_00058216 | NA | 13 | 50044515 | 50082041 | ENSG00000198553 | KCNRG |
| TCONS_00133633 | NA | 2 | 174666238 | 174682916 | ENSG00000138435 | CHRNA1 |
| TCONS_00133633 | NA | 2 | 174666238 | 174682916 | ENSG00000115935 | WIPF1 |
| ENSG00000276337 | AC105429.1 | 16 | 87470370 | 87474370 | ENSG00000103264 | FBXO31 |
| ENSG00000276337 | AC105429.1 | 16 | 87470370 | 87474370 | ENSG00000140941 | MAP1LC3B |
| ENSG00000276337 | AC105429.1 | 16 | 87470370 | 87474370 | ENSG00000140948 | ZCCHC14 |
| ENSG00000232774 | AL355916.1 | 14 | 61570405 | 61658696 | ENSG00000027075 | PRKCH |
| ENSG00000232774 | AL355916.1 | 14 | 61570405 | 61658696 | ENSG00000258989 | AL355916.3 |
| ENSG00000232774 | AL355916.1 | 14 | 61570405 | 61658696 | ENSG00000100644 | HIF1A |
| TCONS_00041570 | NA | 11 | 122174358 | 122367785 | ENSG00000259571 | BLID |
| ENSG00000248690 | HAS2-AS1 | 8 | 121639293 | 121994185 | ENSG00000170961 | HAS2 |
| TCONS_00052185 | NA | 12 | 65921416 | 65948479 | ENSG00000149948 | HMGA2 |
| TCONS_00027870 | NA | 10 | 110474374 | 110495386 | ENSG00000108055 | SMC3 |
| TCONS_00027870 | NA | 10 | 110474374 | 110495386 | ENSG00000138166 | DUSP5 |
| ENSG00000271643 | AC112220.2 | 3 | 33793644 | 33798539 | ENSG00000170248 | PDCD6IP |
| ENSG00000271643 | AC112220.2 | 3 | 33793644 | 33798539 | ENSG00000163539 | CLASP2 |
| ENSG00000253773 | C8orf37-AS1 | 8 | 95204456 | 95811254 | ENSG00000156170 | NDUFAF6 |
| ENSG00000253773 | C8orf37-AS1 | 8 | 95204456 | 95811254 | ENSG00000156172 | C8orf37 |
| ENSG00000253773 | C8orf37-AS1 | 8 | 95204456 | 95811254 | ENSG00000175895 | PLEKHF2 |
| ENSG00000267279 | AC090409.1 | 18 | 61585746 | 61607006 | ENSG00000101542 | CDH20 |
| ENSG00000286553 | AL357075.3 | 6 | 154477982 | 154479593 | ENSG00000153721 | CNKSR3 |
| ENSG00000286553 | AL357075.3 | 6 | 154477982 | 154479593 | ENSG00000288520 | AL357075.5 |
| ENSG00000227482 | AL157702.2 | 9 | 113650956 | 113682855 | ENSG00000138835 | RGS3 |
| ENSG00000253642 | AF279873.3 | 8 | 33604856 | 34039104 | ENSG00000133878 | DUSP26 |
| ENSG00000253642 | AF279873.3 | 8 | 33604856 | 34039104 | ENSG00000133874 | RNF122 |
| ENSG00000253642 | AF279873.3 | 8 | 33604856 | 34039104 | ENSG00000129696 | TTI2 |
| ENSG00000277152 | AC110048.2 | 15 | 66860303 | 66867023 | ENSG00000137834 | SMAD6 |
| TCONS_00024947 | NA | 10 | 10784593 | 10795047 | ENSG00000048740 | CELF2 |
| ENSG00000228261 | ITPRIP-AS1 | 10 | 104323369 | 104327004 | ENSG00000148841 | ITPRIP |
| ENSG00000228261 | ITPRIP-AS1 | 10 | 104323369 | 104327004 | ENSG00000148834 | GSTO1 |
| ENSG00000228261 | ITPRIP-AS1 | 10 | 104323369 | 104327004 | ENSG00000065621 | GSTO2 |
| ENSG00000228261 | ITPRIP-AS1 | 10 | 104323369 | 104327004 | ENSG00000120051 | CFAP58 |
| ENSG00000228261 | ITPRIP-AS1 | 10 | 104323369 | 104327004 | ENSG00000197748 | CFAP43 |
| ENSG00000226194 | LINC02519 | 6 | 169369998 | 169388385 | ENSG00000184465 | WDR27 |
| ENSG00000226194 | LINC02519 | 6 | 169369998 | 169388385 | ENSG00000285733 | AL031315.1 |
| ENSG00000248927 | AC114284.1 | 5 | 120781218 | 120790778 | ENSG00000184838 | PRR16 |
| ENSG00000276724 | AC123768.4 | 15 | 32583612 | 32584312 | ENSG00000232653 | GOLGA8N |
| ENSG00000276724 | AC123768.4 | 15 | 32583612 | 32584312 | ENSG00000166922 | SCG5 |
| ENSG00000276724 | AC123768.4 | 15 | 32583612 | 32584312 | ENSG00000198826 | ARHGAP11A |
| ENSG00000254887 | AC010247.1 | 19 | 42132555 | 42137099 | ENSG00000105732 | ZNF574 |
| ENSG00000254887 | AC010247.1 | 19 | 42132555 | 42137099 | ENSG00000105723 | GSK3A |
| ENSG00000254887 | AC010247.1 | 19 | 42132555 | 42137099 | ENSG00000105737 | GRIK5 |
| ENSG00000254887 | AC010247.1 | 19 | 42132555 | 42137099 | ENSG00000028277 | POU2F2 |
| ENSG00000254887 | AC010247.1 | 19 | 42132555 | 42137099 | ENSG00000167625 | ZNF526 |
| ENSG00000254887 | AC010247.1 | 19 | 42132555 | 42137099 | ENSG00000160570 | DEDD2 |
| ENSG00000254887 | AC010247.1 | 19 | 42132555 | 42137099 | ENSG00000268643 | AC006486.1 |
| ENSG00000250899 | AC125807.2 | 12 | 3041437 | 3044950 | ENSG00000011105 | TSPAN9 |
| ENSG00000250899 | AC125807.2 | 12 | 3041437 | 3044950 | ENSG00000197905 | TEAD4 |
| TCONS_00178849 | NA | 5 | 174825849 | 174826433 | ENSG00000120149 | MSX2 |
| ENSG00000268388 | FENDRR | 16 | 86474529 | 86509099 | ENSG00000103241 | FOXF1 |
| ENSG00000268388 | FENDRR | 16 | 86474529 | 86509099 | ENSG00000176692 | FOXC2 |
| ENSG00000268388 | FENDRR | 16 | 86474529 | 86509099 | ENSG00000103248 | MTHFSD |
| ENSG00000268388 | FENDRR | 16 | 86474529 | 86509099 | ENSG00000176678 | FOXL1 |
| ENSG00000248429 | FAM198B-AS1 | 4 | 158170752 | 158202877 | ENSG00000164125 | GASK1B |
| ENSG00000248429 | FAM198B-AS1 | 4 | 158170752 | 158202877 | ENSG00000164124 | TMEM144 |
| TCONS_00106642 | NA | 19 | 9996066 | 9997401 | ENSG00000080573 | COL5A3 |
| TCONS_00106642 | NA | 19 | 9996066 | 9997401 | ENSG00000080511 | RDH8 |
| TCONS_00106642 | NA | 19 | 9996066 | 9997401 | ENSG00000105088 | OLFM2 |
| TCONS_00106642 | NA | 19 | 9996066 | 9997401 | ENSG00000130812 | ANGPTL6 |
| TCONS_00106642 | NA | 19 | 9996066 | 9997401 | ENSG00000130813 | SHFL |
| TCONS_00066037 | NA | 14 | 71292751 | 71326284 | ENSG00000197555 | SIPA1L1 |
| ENSG00000280109 | PLAC4 | 21 | 41175231 | 41186788 | ENSG00000182240 | BACE2 |
| ENSG00000250295 | RDH10-AS1 | 8 | 73297711 | 73356461 | ENSG00000121039 | RDH10 |
| ENSG00000250295 | RDH10-AS1 | 8 | 73297711 | 73356461 | ENSG00000274443 | C8orf89 |
| ENSG00000250295 | RDH10-AS1 | 8 | 73297711 | 73356461 | ENSG00000147604 | RPL7 |
| ENSG00000250295 | RDH10-AS1 | 8 | 73297711 | 73356461 | ENSG00000040341 | STAU2 |
| ENSG00000259847 | LINC02126 | 16 | 65141751 | 65176713 | ENSG00000140937 | CDH11 |
| ENSG00000251144 | AC113346.1 | 5 | 174820027 | 174826324 | ENSG00000120149 | MSX2 |
| ENSG00000230606 | AC092683.1 | 2 | 97416165 | 97433527 | ENSG00000196912 | ANKRD36B |
| ENSG00000230498 | AL035409.1 | 1 | 77067920 | 77086402 | ENSG00000142892 | PIGK |
| ENSG00000230498 | AL035409.1 | 1 | 77067920 | 77086402 | ENSG00000117069 | ST6GALNAC5 |
| ENSG00000226101 | LINC02097 | 17 | 72072333 | 72103035 | ENSG00000125398 | SOX9 |
| ENSG00000257259 | LINC02388 | 12 | 58565959 | 58781747 | ENSG00000139263 | LRIG3 |
| ENSG00000248206 | AC074194.1 | 4 | 183987669 | 183996680 | ENSG00000173320 | STOX2 |
| ENSG00000248206 | AC074194.1 | 4 | 183987669 | 183996680 | ENSG00000164303 | ENPP6 |
| ENSG00000287576 | AC067930.8 | 8 | 143541648 | 143545128 | ENSG00000179886 | TIGD5 |
| ENSG00000287576 | AC067930.8 | 8 | 143541648 | 143545128 | ENSG00000014164 | ZC3H3 |
| ENSG00000287576 | AC067930.8 | 8 | 143541648 | 143545128 | ENSG00000104522 | TSTA3 |
| ENSG00000287576 | AC067930.8 | 8 | 143541648 | 143545128 | ENSG00000104524 | PYCR3 |
| ENSG00000287576 | AC067930.8 | 8 | 143541648 | 143545128 | ENSG00000104518 | GSDMD |
| ENSG00000287576 | AC067930.8 | 8 | 143541648 | 143545128 | ENSG00000104529 | EEF1D |
| ENSG00000287576 | AC067930.8 | 8 | 143541648 | 143545128 | ENSG00000147813 | NAPRT |
| ENSG00000287576 | AC067930.8 | 8 | 143541648 | 143545128 | ENSG00000183309 | ZNF623 |
| ENSG00000287576 | AC067930.8 | 8 | 143541648 | 143545128 | ENSG00000204839 | MROH6 |
| ENSG00000226200 | SGMS1-AS1 | 10 | 50623466 | 50641451 | ENSG00000204147 | ASAH2B |
| ENSG00000226200 | SGMS1-AS1 | 10 | 50623466 | 50641451 | ENSG00000198964 | SGMS1 |
| ENSG00000259884 | AC025259.3 | 12 | 52058459 | 52059503 | ENSG00000123358 | NR4A1 |
| ENSG00000259884 | AC025259.3 | 12 | 52058459 | 52059503 | ENSG00000161835 | GRASP |
| ENSG00000259884 | AC025259.3 | 12 | 52058459 | 52059503 | ENSG00000135503 | ACVR1B |
| ENSG00000259884 | AC025259.3 | 12 | 52058459 | 52059503 | ENSG00000123395 | ATG101 |
| ENSG00000259884 | AC025259.3 | 12 | 52058459 | 52059503 | ENSG00000284791 | SMIM41 |
| ENSG00000249087 | ZNF436-AS1 | 1 | 23368997 | 23371839 | ENSG00000088280 | ASAP3 |
| ENSG00000249087 | ZNF436-AS1 | 1 | 23368997 | 23371839 | ENSG00000125945 | ZNF436 |
| ENSG00000249087 | ZNF436-AS1 | 1 | 23368997 | 23371839 | ENSG00000204219 | TCEA3 |
| ENSG00000249087 | ZNF436-AS1 | 1 | 23368997 | 23371839 | ENSG00000125944 | HNRNPR |
| ENSG00000287400 | AC093151.8 | 1 | 41241772 | 41338644 | ENSG00000010803 | SCMH1 |
| ENSG00000287400 | AC093151.8 | 1 | 41241772 | 41338644 | ENSG00000204060 | FOXO6 |
| ENSG00000244953 | AC087521.1 | 11 | 43943787 | 43947206 | ENSG00000149084 | HSD17B12 |
| ENSG00000244953 | AC087521.1 | 11 | 43943787 | 43947206 | ENSG00000166199 | ALKBH3 |
| ENSG00000244953 | AC087521.1 | 11 | 43943787 | 43947206 | ENSG00000187479 | C11orf96 |
| ENSG00000260196 | AC124798.1 | 11 | 17380649 | 17383531 | ENSG00000187486 | KCNJ11 |
| ENSG00000260196 | AC124798.1 | 11 | 17380649 | 17383531 | ENSG00000070081 | NUCB2 |
| ENSG00000260196 | AC124798.1 | 11 | 17380649 | 17383531 | ENSG00000188211 | NCR3LG1 |
| ENSG00000260196 | AC124798.1 | 11 | 17380649 | 17383531 | ENSG00000006071 | ABCC8 |
| ENSG00000236017 | ASMTL-AS1 | X | 1401769 | 1414028 | ENSG00000198223 | CSF2RA |
| ENSG00000236017 | ASMTL-AS1 | X | 1401769 | 1414028 | ENSG00000169100 | SLC25A6 |
| ENSG00000236017 | ASMTL-AS1 | X | 1401769 | 1414028 | ENSG00000182162 | P2RY8 |
| ENSG00000236017 | ASMTL-AS1 | X | 1401769 | 1414028 | ENSG00000169093 | ASMTL |
| ENSG00000236017 | ASMTL-AS1 | X | 1401769 | 1414028 | ENSG00000185291 | IL3RA |
| ENSG00000286458 | AC083870.1 | 7 | 134816543 | 134998153 | ENSG00000146856 | AGBL3 |
| ENSG00000286458 | AC083870.1 | 7 | 134816543 | 134998153 | ENSG00000122786 | CALD1 |
| ENSG00000286458 | AC083870.1 | 7 | 134816543 | 134998153 | ENSG00000122783 | CYREN |
| ENSG00000250634 | LINC01182 | 4 | 13654374 | 14003756 | ENSG00000038219 | BOD1L1 |
| ENSG00000214353 | VAC14-AS1 | 16 | 70755035 | 70773251 | ENSG00000103043 | VAC14 |
| ENSG00000214353 | VAC14-AS1 | 16 | 70755035 | 70773251 | ENSG00000157423 | HYDIN |
| ENSG00000214353 | VAC14-AS1 | 16 | 70755035 | 70773251 | ENSG00000157368 | IL34 |
| ENSG00000214353 | VAC14-AS1 | 16 | 70755035 | 70773251 | ENSG00000132613 | MTSS2 |
| ENSG00000224189 | HAGLR | 2 | 176164051 | 176188958 | ENSG00000128645 | HOXD1 |
| ENSG00000224189 | HAGLR | 2 | 176164051 | 176188958 | ENSG00000175879 | HOXD8 |
| ENSG00000224189 | HAGLR | 2 | 176164051 | 176188958 | ENSG00000128652 | HOXD3 |
| ENSG00000224189 | HAGLR | 2 | 176164051 | 176188958 | ENSG00000174279 | EVX2 |
| ENSG00000224189 | HAGLR | 2 | 176164051 | 176188958 | ENSG00000128709 | HOXD9 |
| ENSG00000224189 | HAGLR | 2 | 176164051 | 176188958 | ENSG00000128654 | MTX2 |
| ENSG00000224189 | HAGLR | 2 | 176164051 | 176188958 | ENSG00000128713 | HOXD11 |
| ENSG00000224189 | HAGLR | 2 | 176164051 | 176188958 | ENSG00000170178 | HOXD12 |
| ENSG00000224189 | HAGLR | 2 | 176164051 | 176188958 | ENSG00000170166 | HOXD4 |
| ENSG00000224189 | HAGLR | 2 | 176164051 | 176188958 | ENSG00000128714 | HOXD13 |
| ENSG00000224189 | HAGLR | 2 | 176164051 | 176188958 | ENSG00000128710 | HOXD10 |
| ENSG00000276672 | AL161891.1 | 13 | 33846190 | 33850825 | ENSG00000133119 | RFC3 |
| ENSG00000254102 | AC090136.3 | 8 | 64574306 | 64581921 | ENSG00000180828 | BHLHE22 |
| ENSG00000254102 | AC090136.3 | 8 | 64574306 | 64581921 | ENSG00000172817 | CYP7B1 |
| ENSG00000250343 | STK32A-AS1 | 5 | 147180204 | 147234859 | ENSG00000169302 | STK32A |
| ENSG00000250343 | STK32A-AS1 | 5 | 147180204 | 147234859 | ENSG00000156475 | PPP2R2B |
| ENSG00000218537 | MIF-AS1 | 22 | 23894426 | 23898930 | ENSG00000276950 | GSTT4 |
| ENSG00000218537 | MIF-AS1 | 22 | 23894426 | 23898930 | ENSG00000240972 | MIF |
| ENSG00000218537 | MIF-AS1 | 22 | 23894426 | 23898930 | ENSG00000251357 | AP000350.4 |
| ENSG00000218537 | MIF-AS1 | 22 | 23894426 | 23898930 | ENSG00000285762 | AC253536.7 |
| ENSG00000218537 | MIF-AS1 | 22 | 23894426 | 23898930 | ENSG00000133433 | GSTT2B |
| ENSG00000218537 | MIF-AS1 | 22 | 23894426 | 23898930 | ENSG00000099974 | DDTL |
| ENSG00000218537 | MIF-AS1 | 22 | 23894426 | 23898930 | ENSG00000099958 | DERL3 |
| ENSG00000218537 | MIF-AS1 | 22 | 23894426 | 23898930 | ENSG00000099956 | SMARCB1 |
| ENSG00000218537 | MIF-AS1 | 22 | 23894426 | 23898930 | ENSG00000133460 | SLC2A11 |
| ENSG00000218537 | MIF-AS1 | 22 | 23894426 | 23898930 | ENSG00000099977 | DDT |
| ENSG00000261490 | AC005674.2 | 4 | 10068089 | 10073019 | ENSG00000109667 | SLC2A9 |
| ENSG00000261490 | AC005674.2 | 4 | 10068089 | 10073019 | ENSG00000071127 | WDR1 |
| ENSG00000225956 | AL050320.1 | 20 | 13244064 | 13245369 | ENSG00000172296 | SPTLC3 |
| ENSG00000225956 | AL050320.1 | 20 | 13244064 | 13245369 | ENSG00000101230 | ISM1 |
| ENSG00000249790 | AC092490.1 | 12 | 8788253 | 8795789 | ENSG00000166532 | RIMKLB |
| ENSG00000249790 | AC092490.1 | 12 | 8788253 | 8795789 | ENSG00000166535 | A2ML1 |
| ENSG00000226496 | LINC00323 | 21 | 41141493 | 41148198 | ENSG00000182240 | BACE2 |
| ENSG00000284669 | AC092053.3 | 3 | 39148281 | 39172952 | ENSG00000168026 | TTC21A |
| ENSG00000284669 | AC092053.3 | 3 | 39148281 | 39172952 | ENSG00000168329 | CX3CR1 |
| ENSG00000284669 | AC092053.3 | 3 | 39148281 | 39172952 | ENSG00000168334 | XIRP1 |
| ENSG00000284669 | AC092053.3 | 3 | 39148281 | 39172952 | ENSG00000168356 | SCN11A |
| ENSG00000284669 | AC092053.3 | 3 | 39148281 | 39172952 | ENSG00000144655 | CSRNP1 |
| ENSG00000284669 | AC092053.3 | 3 | 39148281 | 39172952 | ENSG00000114742 | WDR48 |
| ENSG00000284669 | AC092053.3 | 3 | 39148281 | 39172952 | ENSG00000114745 | GORASP1 |
| ENSG00000231999 | LRRC8C-DT | 1 | 89581291 | 89632916 | ENSG00000171488 | LRRC8C |
| ENSG00000231999 | LRRC8C-DT | 1 | 89581291 | 89632916 | ENSG00000271949 | AC093423.3 |
| ENSG00000231999 | LRRC8C-DT | 1 | 89581291 | 89632916 | ENSG00000197147 | LRRC8B |
| ENSG00000246898 | LINC00920 | 16 | 66408524 | 66412135 | ENSG00000166546 | BEAN1 |
| ENSG00000246898 | LINC00920 | 16 | 66408524 | 66412135 | ENSG00000166548 | TK2 |
| ENSG00000246898 | LINC00920 | 16 | 66408524 | 66412135 | ENSG00000179776 | CDH5 |
| ENSG00000246898 | LINC00920 | 16 | 66408524 | 66412135 | ENSG00000260851 | AC010542.3 |
| ENSG00000281398 | SNHG4 | 5 | 139274102 | 139284899 | ENSG00000120727 | PAIP2 |
| ENSG00000281398 | SNHG4 | 5 | 139274102 | 139284899 | ENSG00000015479 | MATR3 |
| ENSG00000281398 | SNHG4 | 5 | 139274102 | 139284899 | ENSG00000120725 | SIL1 |
| ENSG00000281398 | SNHG4 | 5 | 139274102 | 139284899 | ENSG00000170482 | SLC23A1 |
| ENSG00000281398 | SNHG4 | 5 | 139274102 | 139284899 | ENSG00000280987 | MATR3 |
| ENSG00000228060 | PABPC4-AS1 | 1 | 39565052 | 39573860 | ENSG00000090621 | PABPC4 |
| ENSG00000228060 | PABPC4-AS1 | 1 | 39565052 | 39573860 | ENSG00000183682 | BMP8A |
| ENSG00000228060 | PABPC4-AS1 | 1 | 39565052 | 39573860 | ENSG00000127603 | MACF1 |
| ENSG00000228060 | PABPC4-AS1 | 1 | 39565052 | 39573860 | ENSG00000116981 | NT5C1A |
| ENSG00000228060 | PABPC4-AS1 | 1 | 39565052 | 39573860 | ENSG00000163909 | HEYL |
| ENSG00000256083 | AC090673.1 | 12 | 65934777 | 65948479 | ENSG00000149948 | HMGA2 |
| ENSG00000247095 | MIR210HG | 11 | 565660 | 568457 | ENSG00000185507 | IRF7 |
| ENSG00000247095 | MIR210HG | 11 | 565660 | 568457 | ENSG00000161328 | LRRC56 |
| ENSG00000247095 | MIR210HG | 11 | 565660 | 568457 | ENSG00000070047 | PHRF1 |
| ENSG00000247095 | MIR210HG | 11 | 565660 | 568457 | ENSG00000099834 | CDHR5 |
| ENSG00000247095 | MIR210HG | 11 | 565660 | 568457 | ENSG00000070031 | SCT |
| ENSG00000247095 | MIR210HG | 11 | 565660 | 568457 | ENSG00000099849 | RASSF7 |
| ENSG00000247095 | MIR210HG | 11 | 565660 | 568457 | ENSG00000185522 | LMNTD2 |
| ENSG00000247095 | MIR210HG | 11 | 565660 | 568457 | ENSG00000023191 | RNH1 |
| ENSG00000247095 | MIR210HG | 11 | 565660 | 568457 | ENSG00000174915 | PTDSS2 |
| ENSG00000247095 | MIR210HG | 11 | 565660 | 568457 | ENSG00000069696 | DRD4 |
| ENSG00000247095 | MIR210HG | 11 | 565660 | 568457 | ENSG00000177030 | DEAF1 |
| ENSG00000247095 | MIR210HG | 11 | 565660 | 568457 | ENSG00000174775 | HRAS |
| ENSG00000249628 | LINC00942 | 12 | 1500525 | 1504424 | ENSG00000111186 | WNT5B |
| ENSG00000249628 | LINC00942 | 12 | 1500525 | 1504424 | ENSG00000171823 | FBXL14 |
| ENSG00000249628 | LINC00942 | 12 | 1500525 | 1504424 | ENSG00000082805 | ERC1 |
| ENSG00000225684 | FAM225B | 9 | 113102117 | 113111543 | ENSG00000119321 | FKBP15 |
| ENSG00000225684 | FAM225B | 9 | 113102117 | 113111543 | ENSG00000136867 | SLC31A2 |
| ENSG00000225684 | FAM225B | 9 | 113102117 | 113111543 | ENSG00000136866 | ZFP37 |
| ENSG00000225684 | FAM225B | 9 | 113102117 | 113111543 | ENSG00000285447 | ZNF883 |
| ENSG00000279082 | LINC01727 | 20 | 21569976 | 21666621 | ENSG00000125820 | NKX2-2 |
| ENSG00000279082 | LINC01727 | 20 | 21569976 | 21666621 | ENSG00000125813 | PAX1 |
| TCONS_00042146 | NA | 12 | 1484728 | 1507890 | ENSG00000111186 | WNT5B |
| TCONS_00042146 | NA | 12 | 1484728 | 1507890 | ENSG00000171823 | FBXL14 |
| TCONS_00042146 | NA | 12 | 1484728 | 1507890 | ENSG00000082805 | ERC1 |
| TCONS_00143645 | NA | 21 | 28438442 | 28772221 | ENSG00000156239 | N6AMT1 |
| ENSG00000285664 | AL139317.5 | 14 | 52791756 | 52930185 | ENSG00000100522 | GNPNAT1 |
| ENSG00000285664 | AL139317.5 | 14 | 52791756 | 52930185 | ENSG00000198252 | STYX |
| ENSG00000285664 | AL139317.5 | 14 | 52791756 | 52930185 | ENSG00000197930 | ERO1A |
| ENSG00000285664 | AL139317.5 | 14 | 52791756 | 52930185 | ENSG00000100519 | PSMC6 |
| ENSG00000285664 | AL139317.5 | 14 | 52791756 | 52930185 | ENSG00000073712 | FERMT2 |
| ENSG00000257894 | AC027288.3 | 12 | 79341205 | 79503396 | ENSG00000177425 | PAWR |
| ENSG00000257894 | AC027288.3 | 12 | 79341205 | 79503396 | ENSG00000067715 | SYT1 |
| TCONS_00015778 | NA | 1 | 115311505 | 115338236 | ENSG00000134259 | NGF |
| ENSG00000253875 | AC013643.2 | 8 | 27732915 | 27758552 | ENSG00000147419 | CCDC25 |
| ENSG00000253875 | AC013643.2 | 8 | 27732915 | 27758552 | ENSG00000168078 | PBK |
| ENSG00000253875 | AC013643.2 | 8 | 27732915 | 27758552 | ENSG00000168077 | SCARA3 |
| ENSG00000253875 | AC013643.2 | 8 | 27732915 | 27758552 | ENSG00000171320 | ESCO2 |
| ENSG00000259065 | AC005520.2 | 14 | 73787355 | 73803300 | ENSG00000258653 | AC005520.1 |
| ENSG00000259065 | AC005520.2 | 14 | 73787355 | 73803300 | ENSG00000176903 | PNMA1 |
| ENSG00000259065 | AC005520.2 | 14 | 73787355 | 73803300 | ENSG00000156030 | ELMSAN1 |
| ENSG00000259065 | AC005520.2 | 14 | 73787355 | 73803300 | ENSG00000140043 | PTGR2 |
| ENSG00000259065 | AC005520.2 | 14 | 73787355 | 73803300 | ENSG00000119661 | DNAL1 |
| ENSG00000259065 | AC005520.2 | 14 | 73787355 | 73803300 | ENSG00000119725 | ZNF410 |
| TCONS_00205317 | NA | 7 | 130998535 | 131019970 | ENSG00000128585 | MKLN1 |
| ENSG00000250659 | AP001363.1 | 11 | 62537312 | 62542018 | ENSG00000089597 | GANAB |
| ENSG00000250659 | AP001363.1 | 11 | 62537312 | 62542018 | ENSG00000124942 | AHNAK |
| ENSG00000250659 | AP001363.1 | 11 | 62537312 | 62542018 | ENSG00000149489 | ROM1 |
| ENSG00000250659 | AP001363.1 | 11 | 62537312 | 62542018 | ENSG00000149499 | EML3 |
| ENSG00000250659 | AP001363.1 | 11 | 62537312 | 62542018 | ENSG00000255508 | AP002990.1 |
| ENSG00000250659 | AP001363.1 | 11 | 62537312 | 62542018 | ENSG00000149016 | TUT1 |
| ENSG00000250659 | AP001363.1 | 11 | 62537312 | 62542018 | ENSG00000149541 | B3GAT3 |
| ENSG00000250659 | AP001363.1 | 11 | 62537312 | 62542018 | ENSG00000149480 | MTA2 |
| ENSG00000250659 | AP001363.1 | 11 | 62537312 | 62542018 | ENSG00000254772 | EEF1G |
| ENSG00000225383 | SFTA1P | 10 | 10784437 | 10795047 | ENSG00000048740 | CELF2 |
| ENSG00000284606 | AC105233.5 | 8 | 8059572 | 8199973 | ENSG00000225327 | USP17L3 |
| ENSG00000284606 | AC105233.5 | 8 | 8059572 | 8199973 | ENSG00000237038 | USP17L8 |
| ENSG00000253522 | MIR3142HG | 5 | 160438594 | 160487426 | ENSG00000145861 | C1QTNF2 |
| ENSG00000253522 | MIR3142HG | 5 | 160438594 | 160487426 | ENSG00000164609 | SLU7 |
| ENSG00000253522 | MIR3142HG | 5 | 160438594 | 160487426 | ENSG00000118322 | ATP10B |
| ENSG00000253522 | MIR3142HG | 5 | 160438594 | 160487426 | ENSG00000164611 | PTTG1 |
| ENSG00000253522 | MIR3142HG | 5 | 160438594 | 160487426 | ENSG00000221886 | ZBED8 |
| ENSG00000253522 | MIR3142HG | 5 | 160438594 | 160487426 | ENSG00000135083 | CCNJL |
| ENSG00000267943 | AC010328.1 | 19 | 53007512 | 53013180 | ENSG00000180257 | ZNF816 |
| ENSG00000267943 | AC010328.1 | 19 | 53007512 | 53013180 | ENSG00000170954 | ZNF415 |
| ENSG00000267943 | AC010328.1 | 19 | 53007512 | 53013180 | ENSG00000268964 | ERVV-2 |
| ENSG00000267943 | AC010328.1 | 19 | 53007512 | 53013180 | ENSG00000213793 | ZNF888 |
| ENSG00000267943 | AC010328.1 | 19 | 53007512 | 53013180 | ENSG00000269526 | ERVV-1 |
| ENSG00000267943 | AC010328.1 | 19 | 53007512 | 53013180 | ENSG00000221874 | ZNF816-ZNF321P |
| ENSG00000267943 | AC010328.1 | 19 | 53007512 | 53013180 | ENSG00000170949 | ZNF160 |
| ENSG00000224671 | AC105940.1 | 1 | 203144694 | 203152579 | ENSG00000163485 | ADORA1 |
| ENSG00000224671 | AC105940.1 | 1 | 203144694 | 203152579 | ENSG00000133055 | MYBPH |
| ENSG00000224671 | AC105940.1 | 1 | 203144694 | 203152579 | ENSG00000133063 | CHIT1 |
| ENSG00000224671 | AC105940.1 | 1 | 203144694 | 203152579 | ENSG00000122180 | MYOG |
| ENSG00000224671 | AC105940.1 | 1 | 203144694 | 203152579 | ENSG00000143847 | PPFIA4 |
| ENSG00000224671 | AC105940.1 | 1 | 203144694 | 203152579 | ENSG00000133048 | CHI3L1 |
| ENSG00000273828 | AL133227.1 | 20 | 46364551 | 46397994 | ENSG00000149654 | CDH22 |
| ENSG00000273828 | AL133227.1 | 20 | 46364551 | 46397994 | ENSG00000080189 | SLC35C2 |
| ENSG00000273828 | AL133227.1 | 20 | 46364551 | 46397994 | ENSG00000062598 | ELMO2 |
| ENSG00000286463 | AP002755.1 | 11 | 114159791 | 114161166 | ENSG00000166741 | NNMT |
| ENSG00000286463 | AP002755.1 | 11 | 114159791 | 114161166 | ENSG00000109906 | ZBTB16 |
| ENSG00000281392 | AC107204.1 | 3 | 87089129 | 87158363 | ENSG00000083937 | CHMP2B |
| ENSG00000281392 | AC107204.1 | 3 | 87089129 | 87158363 | ENSG00000206538 | VGLL3 |
| TCONS_00016280 | NA | 1 | 149084156 | 149103875 | ENSG00000269713 | NBPF9 |
| TCONS_00016280 | NA | 1 | 149084156 | 149103875 | ENSG00000178104 | PDE4DIP |
| ENSG00000277159 | AL139384.2 | 13 | 112602828 | 112606417 | ENSG00000126216 | TUBGCP3 |
| ENSG00000277159 | AL139384.2 | 13 | 112602828 | 112606417 | ENSG00000068650 | ATP11A |
| ENSG00000259146 | AC005476.2 | 14 | 71292729 | 71321882 | ENSG00000197555 | SIPA1L1 |
| ENSG00000286429 | BX004807.1 | 1 | 63487957 | 63508204 | ENSG00000203965 | EFCAB7 |
| ENSG00000286429 | BX004807.1 | 1 | 63487957 | 63508204 | ENSG00000088035 | ALG6 |
| ENSG00000286429 | BX004807.1 | 1 | 63487957 | 63508204 | ENSG00000079739 | PGM1 |
| ENSG00000286429 | BX004807.1 | 1 | 63487957 | 63508204 | ENSG00000142856 | ITGB3BP |
| ENSG00000261512 | AC092368.3 | 16 | 46622861 | 46624451 | ENSG00000091651 | ORC6 |
| ENSG00000261512 | AC092368.3 | 16 | 46622861 | 46624451 | ENSG00000171241 | SHCBP1 |
| ENSG00000261512 | AC092368.3 | 16 | 46622861 | 46624451 | ENSG00000140795 | MYLK3 |
| ENSG00000261512 | AC092368.3 | 16 | 46622861 | 46624451 | ENSG00000069329 | VPS35 |
| ENSG00000263438 | LINC01919 | 18 | 53568439 | 53629990 | ENSG00000187323 | DCC |
| ENSG00000273374 | AC069222.1 | 3 | 99802699 | 99806058 | ENSG00000144810 | COL8A1 |
| ENSG00000273374 | AC069222.1 | 3 | 99802699 | 99806058 | ENSG00000184220 | CMSS1 |
| ENSG00000273374 | AC069222.1 | 3 | 99802699 | 99806058 | ENSG00000168386 | FILIP1L |
| ENSG00000204362 | LINC02783 | 1 | 17189783 | 17197617 | ENSG00000142623 | PADI1 |
| ENSG00000204362 | LINC02783 | 1 | 17189783 | 17197617 | ENSG00000117115 | PADI2 |
| ENSG00000204362 | LINC02783 | 1 | 17189783 | 17197617 | ENSG00000142619 | PADI3 |
| ENSG00000247134 | AC090204.1 | 8 | 32927913 | 33045445 | ENSG00000157168 | NRG1 |
| ENSG00000205414 | AC007608.1 | 16 | 50606076 | 50613684 | ENSG00000167207 | NOD2 |
| ENSG00000205414 | AC007608.1 | 16 | 50606076 | 50613684 | ENSG00000140807 | NKD1 |
| ENSG00000205414 | AC007608.1 | 16 | 50606076 | 50613684 | ENSG00000167208 | SNX20 |
| ENSG00000231373 | GNA14-AS1 | 9 | 77456295 | 77526697 | ENSG00000197969 | VPS13A |
| ENSG00000231373 | GNA14-AS1 | 9 | 77456295 | 77526697 | ENSG00000156049 | GNA14 |
| TCONS_00181987 | NA | 5 | 111755357 | 111770032 | ENSG00000134986 | NREP |
| ENSG00000227733 | AC239809.3 | 1 | 148159213 | 148255012 | ENSG00000263956 | NBPF11 |
| ENSG00000276851 | AC002401.4 | 17 | 50094065 | 50094647 | ENSG00000108823 | SGCA |
| ENSG00000276851 | AC002401.4 | 17 | 50094065 | 50094647 | ENSG00000108821 | COL1A1 |
| ENSG00000276851 | AC002401.4 | 17 | 50094065 | 50094647 | ENSG00000167100 | SAMD14 |
| ENSG00000276851 | AC002401.4 | 17 | 50094065 | 50094647 | ENSG00000005882 | PDK2 |
| ENSG00000276851 | AC002401.4 | 17 | 50094065 | 50094647 | ENSG00000064195 | DLX3 |
| ENSG00000276851 | AC002401.4 | 17 | 50094065 | 50094647 | ENSG00000108819 | PPP1R9B |
| ENSG00000276851 | AC002401.4 | 17 | 50094065 | 50094647 | ENSG00000005884 | ITGA3 |
| ENSG00000203721 | LINC00862 | 1 | 200253419 | 200400705 | ENSG00000162702 | ZNF281 |
| ENSG00000203721 | LINC00862 | 1 | 200253419 | 200400705 | ENSG00000116833 | NR5A2 |
| TCONS_00207646 | NA | 8 | 32927945 | 33045445 | ENSG00000157168 | NRG1 |
| ENSG00000254554 | AC080023.1 | 11 | 10302657 | 10303704 | ENSG00000133812 | SBF2 |
| ENSG00000254554 | AC080023.1 | 11 | 10302657 | 10303704 | ENSG00000148926 | ADM |
| ENSG00000254554 | AC080023.1 | 11 | 10302657 | 10303704 | ENSG00000133805 | AMPD3 |
| ENSG00000285603 | AL035693.1 | 6 | 2227803 | 2230261 | ENSG00000112699 | GMDS |
| ENSG00000283982 | AL445623.2 | 9 | 23500691 | 23672387 | ENSG00000107105 | ELAVL2 |
| ENSG00000287302 | AL133477.3 | 9 | 96419722 | 96421129 | ENSG00000130956 | HABP4 |
| ENSG00000287302 | AL133477.3 | 9 | 96419722 | 96421129 | ENSG00000130958 | SLC35D2 |
| ENSG00000287302 | AL133477.3 | 9 | 96419722 | 96421129 | ENSG00000081377 | CDC14B |
| ENSG00000287302 | AL133477.3 | 9 | 96419722 | 96421129 | ENSG00000285269 | AL160269.1 |
| ENSG00000287302 | AL133477.3 | 9 | 96419722 | 96421129 | ENSG00000165244 | ZNF367 |
| ENSG00000259946 | BX005019.1 | 1 | 97967005 | 97968814 | ENSG00000188641 | DPYD |
| ENSG00000257681 | AC025265.1 | 12 | 103746315 | 103768858 | ENSG00000111696 | NT5DC3 |
| ENSG00000257681 | AC025265.1 | 12 | 103746315 | 103768858 | ENSG00000136011 | STAB2 |
| ENSG00000285336 | AC108734.4 | 3 | 180707589 | 180871005 | ENSG00000284862 | CCDC39 |
| ENSG00000285336 | AC108734.4 | 3 | 180707589 | 180871005 | ENSG00000163728 | TTC14 |
| ENSG00000285336 | AC108734.4 | 3 | 180707589 | 180871005 | ENSG00000114416 | FXR1 |
| ENSG00000265907 | AP000919.2 | 18 | 2948238 | 2960756 | ENSG00000132205 | EMILIN2 |
| ENSG00000265907 | AP000919.2 | 18 | 2948238 | 2960756 | ENSG00000101577 | LPIN2 |
| ENSG00000280639 | LINC02204 | 15 | 70570958 | 70586606 | ENSG00000137831 | UACA |
| ENSG00000255921 | AC026310.2 | 12 | 24949163 | 24960158 | ENSG00000060982 | BCAT1 |
| ENSG00000255921 | AC026310.2 | 12 | 24949163 | 24960158 | ENSG00000118308 | LRMP |
| ENSG00000286593 | AP006261.1 | 18 | 14404551 | 14430923 | ENSG00000183206 | POTEC |
| ENSG00000286473 | AC133485.7 | 16 | 32126432 | 32132724 | ENSG00000270472 | IGHV3OR16-9 |
| ENSG00000230563 | AL121757.1 | 20 | 5445838 | 5475483 | ENSG00000125772 | GPCPD1 |
| ENSG00000232677 | LINC00665 | 19 | 36313067 | 36331770 | ENSG00000196357 | ZNF565 |
| ENSG00000232677 | LINC00665 | 19 | 36313067 | 36331770 | ENSG00000167635 | ZNF146 |
| ENSG00000232677 | LINC00665 | 19 | 36313067 | 36331770 | ENSG00000181007 | ZFP82 |
| ENSG00000232677 | LINC00665 | 19 | 36313067 | 36331770 | ENSG00000142065 | ZFP14 |
| ENSG00000233968 | AL157895.1 | 10 | 19710328 | 19728550 | ENSG00000120594 | PLXDC2 |
| ENSG00000233968 | AL157895.1 | 10 | 19710328 | 19728550 | ENSG00000204740 | MALRD1 |
| TCONS_00072469 | NA | 15 | 28714116 | 28727164 | ENSG00000188626 | GOLGA8M |
| ENSG00000259426 | AC027237.3 | 15 | 69396904 | 69415029 | ENSG00000137819 | PAQR5 |
| ENSG00000259426 | AC027237.3 | 15 | 69396904 | 69415029 | ENSG00000137818 | RPLP1 |
| ENSG00000259426 | AC027237.3 | 15 | 69396904 | 69415029 | ENSG00000137807 | KIF23 |
| ENSG00000229154 | KCNQ5-AS1 | 6 | 73130646 | 73143514 | ENSG00000256980 | KHDC1L |
| ENSG00000229154 | KCNQ5-AS1 | 6 | 73130646 | 73143514 | ENSG00000243501 | AL365232.1 |
| ENSG00000229154 | KCNQ5-AS1 | 6 | 73130646 | 73143514 | ENSG00000185760 | KCNQ5 |
| ENSG00000229154 | KCNQ5-AS1 | 6 | 73130646 | 73143514 | ENSG00000135314 | KHDC1 |
| ENSG00000285644 | AC108448.3 | 11 | 3057538 | 3064707 | ENSG00000110619 | CARS |
| ENSG00000285644 | AC108448.3 | 11 | 3057538 | 3064707 | ENSG00000021762 | OSBPL5 |
| ENSG00000285644 | AC108448.3 | 11 | 3057538 | 3064707 | ENSG00000205531 | NAP1L4 |
| ENSG00000236671 | PRKG1-AS1 | 10 | 52230398 | 52314507 | ENSG00000107984 | DKK1 |
| ENSG00000236671 | PRKG1-AS1 | 10 | 52230398 | 52314507 | ENSG00000185532 | PRKG1 |
| ENSG00000260025 | CRIM1-DT | 2 | 36354749 | 36355114 | ENSG00000150938 | CRIM1 |
| TCONS_00110331 | NA | 19 | 45079258 | 45088088 | ENSG00000179846 | NKPD1 |
| TCONS_00110331 | NA | 19 | 45079258 | 45088088 | ENSG00000104859 | CLASRP |
| TCONS_00110331 | NA | 19 | 45079258 | 45088088 | ENSG00000007047 | MARK4 |
| TCONS_00110331 | NA | 19 | 45079258 | 45088088 | ENSG00000142252 | GEMIN7 |
| TCONS_00110331 | NA | 19 | 45079258 | 45088088 | ENSG00000104856 | RELB |
| TCONS_00110331 | NA | 19 | 45079258 | 45088088 | ENSG00000189114 | BLOC1S3 |
| TCONS_00110331 | NA | 19 | 45079258 | 45088088 | ENSG00000104866 | PPP1R37 |
| TCONS_00110331 | NA | 19 | 45079258 | 45088088 | ENSG00000007255 | TRAPPC6A |
| TCONS_00110331 | NA | 19 | 45079258 | 45088088 | ENSG00000170684 | ZNF296 |
| TCONS_00110331 | NA | 19 | 45079258 | 45088088 | ENSG00000104853 | CLPTM1 |
| ENSG00000258730 | ITPK1-AS1 | 14 | 93067452 | 93072152 | ENSG00000100605 | ITPK1 |
| ENSG00000235660 | LINC00345 | 13 | 52482804 | 52489216 | ENSG00000136100 | VPS36 |
| ENSG00000235660 | LINC00345 | 13 | 52482804 | 52489216 | ENSG00000136114 | THSD1 |
| ENSG00000235660 | LINC00345 | 13 | 52482804 | 52489216 | ENSG00000136108 | CKAP2 |
| ENSG00000276075 | AC027682.6 | 16 | 67517862 | 67528675 | ENSG00000102974 | CTCF |
| ENSG00000276075 | AC027682.6 | 16 | 67517862 | 67528675 | ENSG00000159720 | ATP6V0D1 |
| ENSG00000276075 | AC027682.6 | 16 | 67517862 | 67528675 | ENSG00000039523 | RIPOR1 |
| ENSG00000276075 | AC027682.6 | 16 | 67517862 | 67528675 | ENSG00000176387 | HSD11B2 |
| ENSG00000276075 | AC027682.6 | 16 | 67517862 | 67528675 | ENSG00000159723 | AGRP |
| ENSG00000235731 | LINC02250 | 15 | 25456271 | 25578813 | ENSG00000206190 | ATP10A |
| ENSG00000235731 | LINC02250 | 15 | 25456271 | 25578813 | ENSG00000114062 | UBE3A |
| ENSG00000274031 | AC092140.2 | 16 | 56465642 | 56466162 | ENSG00000125124 | BBS2 |
| ENSG00000274031 | AC092140.2 | 16 | 56465642 | 56466162 | ENSG00000159461 | AMFR |
| ENSG00000274031 | AC092140.2 | 16 | 56465642 | 56466162 | ENSG00000167005 | NUDT21 |
| ENSG00000274031 | AC092140.2 | 16 | 56465642 | 56466162 | ENSG00000102891 | MT4 |
| ENSG00000274031 | AC092140.2 | 16 | 56465642 | 56466162 | ENSG00000087263 | OGFOD1 |
| ENSG00000213057 | C1orf220 | 1 | 178542752 | 178548889 | ENSG00000075391 | RASAL2 |
| ENSG00000213057 | C1orf220 | 1 | 178542752 | 178548889 | ENSG00000240021 | TEX35 |
| ENSG00000213057 | C1orf220 | 1 | 178542752 | 178548889 | ENSG00000188585 | CLEC20A |
| ENSG00000244564 | PRICKLE2-DT | 3 | 64445231 | 64456070 | ENSG00000163638 | ADAMTS9 |
| ENSG00000244564 | PRICKLE2-DT | 3 | 64445231 | 64456070 | ENSG00000163637 | PRICKLE2 |
| ENSG00000257438 | AC011595.1 | 12 | 106103163 | 106106165 | ENSG00000074590 | NUAK1 |
| ENSG00000283828 | AL137002.2 | 13 | 113149432 | 113154002 | ENSG00000126217 | MCF2L |
| ENSG00000283828 | AL137002.2 | 13 | 113149432 | 113154002 | ENSG00000139842 | CUL4A |
| ENSG00000283828 | AL137002.2 | 13 | 113149432 | 113154002 | ENSG00000126226 | PCID2 |
| ENSG00000283828 | AL137002.2 | 13 | 113149432 | 113154002 | ENSG00000057593 | F7 |
| ENSG00000283828 | AL137002.2 | 13 | 113149432 | 113154002 | ENSG00000126218 | F10 |
| ENSG00000283828 | AL137002.2 | 13 | 113149432 | 113154002 | ENSG00000126231 | PROZ |
| ENSG00000257835 | AC073591.1 | 12 | 77379770 | 77390869 | ENSG00000067798 | NAV3 |
| ENSG00000261114 | AC012181.1 | 16 | 56941028 | 56941726 | ENSG00000102900 | NUP93 |
| ENSG00000261114 | AC012181.1 | 16 | 56941028 | 56941726 | ENSG00000087237 | CETP |
| ENSG00000261114 | AC012181.1 | 16 | 56941028 | 56941726 | ENSG00000140853 | NLRC5 |
| ENSG00000261114 | AC012181.1 | 16 | 56941028 | 56941726 | ENSG00000051108 | HERPUD1 |
| ENSG00000261114 | AC012181.1 | 16 | 56941028 | 56941726 | ENSG00000070915 | SLC12A3 |
| ENSG00000236039 | AC019117.1 | 7 | 17405479 | 17558909 | ENSG00000106546 | AHR |
| ENSG00000236039 | AC019117.1 | 7 | 17405479 | 17558909 | ENSG00000283321 | AC019117.3 |
| ENSG00000198491 | AC007920.1 | 3 | 187197090 | 187207629 | ENSG00000175077 | RTP1 |
| ENSG00000198491 | AC007920.1 | 3 | 187197090 | 187207629 | ENSG00000127241 | MASP1 |
| ENSG00000198491 | AC007920.1 | 3 | 187197090 | 187207629 | ENSG00000163923 | RPL39L |
| ENSG00000175772 | LINC01106 | 2 | 110375138 | 110384442 | ENSG00000256671 | LIMS4 |
| ENSG00000175772 | LINC01106 | 2 | 110375138 | 110384442 | ENSG00000257207 | AC112229.3 |
| TCONS_00128237 | NA | 2 | 9568364 | 9576294 | ENSG00000134308 | YWHAQ |
| TCONS_00128237 | NA | 2 | 9568364 | 9576294 | ENSG00000151694 | ADAM17 |
| TCONS_00128237 | NA | 2 | 9568364 | 9576294 | ENSG00000119203 | CPSF3 |
| TCONS_00128237 | NA | 2 | 9568364 | 9576294 | ENSG00000134330 | IAH1 |
| ENSG00000236449 | AC010894.3 | 2 | 174547141 | 174776720 | ENSG00000163328 | GPR155 |
| ENSG00000236449 | AC010894.3 | 2 | 174547141 | 174776720 | ENSG00000138435 | CHRNA1 |
| ENSG00000236449 | AC010894.3 | 2 | 174547141 | 174776720 | ENSG00000115935 | WIPF1 |
| ENSG00000236449 | AC010894.3 | 2 | 174547141 | 174776720 | ENSG00000128656 | CHN1 |
| ENSG00000226445 | BX322234.1 | 6 | 169213254 | 169245773 | ENSG00000186340 | THBS2 |
| ENSG00000253967 | AC022730.4 | 8 | 70471134 | 70485687 | ENSG00000067167 | TRAM1 |
| ENSG00000253967 | AC022730.4 | 8 | 70471134 | 70485687 | ENSG00000140396 | NCOA2 |
| ENSG00000237220 | AC104777.2 | 2 | 150566134 | 150568080 | ENSG00000115963 | RND3 |
| ENSG00000228624 | HDAC2-AS2 | 6 | 113969701 | 114471705 | ENSG00000249853 | HS3ST5 |
| ENSG00000228624 | HDAC2-AS2 | 6 | 113969701 | 114471705 | ENSG00000196591 | HDAC2 |
| TCONS_00204592 | NA | 7 | 104894585 | 105013424 | ENSG00000187416 | LHFPL3 |
| TCONS_00204592 | NA | 7 | 104894585 | 105013424 | ENSG00000005483 | KMT2E |
| TCONS_00204592 | NA | 7 | 104894585 | 105013424 | ENSG00000135250 | SRPK2 |
| ENSG00000261386 | AC027682.4 | 16 | 67549214 | 67563958 | ENSG00000159753 | CARMIL2 |
| ENSG00000261386 | AC027682.4 | 16 | 67549214 | 67563958 | ENSG00000102974 | CTCF |
| ENSG00000261386 | AC027682.4 | 16 | 67549214 | 67563958 | ENSG00000102977 | ACD |
| ENSG00000261386 | AC027682.4 | 16 | 67549214 | 67563958 | ENSG00000159720 | ATP6V0D1 |
| ENSG00000261386 | AC027682.4 | 16 | 67549214 | 67563958 | ENSG00000039523 | RIPOR1 |
| ENSG00000261386 | AC027682.4 | 16 | 67549214 | 67563958 | ENSG00000159723 | AGRP |
| ENSG00000261386 | AC027682.4 | 16 | 67549214 | 67563958 | ENSG00000102981 | PARD6A |
| ENSG00000261386 | AC027682.4 | 16 | 67549214 | 67563958 | ENSG00000124074 | ENKD1 |
| ENSG00000250938 | AC073475.1 | 4 | 120066958 | 120564789 | ENSG00000164109 | MAD2L1 |
| ENSG00000278175 | GLIDR | 9 | 39748514 | 39810097 | ENSG00000283886 | BX664615.2 |
| ENSG00000251139 | AC084871.1 | 4 | 184813619 | 184821300 | ENSG00000151726 | ACSL1 |
| ENSG00000251139 | AC084871.1 | 4 | 184813619 | 184821300 | ENSG00000151725 | CENPU |
| ENSG00000278981 | AC079298.1 | 4 | 154235980 | 154237598 | ENSG00000197410 | DCHS2 |
| TCONS_00201564 | NA | 7 | 17434477 | 17558909 | ENSG00000106546 | AHR |
| TCONS_00201564 | NA | 7 | 17434477 | 17558909 | ENSG00000283321 | AC019117.3 |
| ENSG00000234091 | AL157392.2 | 10 | 13729383 | 13756200 | ENSG00000151474 | FRMD4A |
| ENSG00000234091 | AL157392.2 | 10 | 13729383 | 13756200 | ENSG00000282246 | AL157392.5 |
| ENSG00000234091 | AL157392.2 | 10 | 13729383 | 13756200 | ENSG00000165630 | PRPF18 |
| ENSG00000286707 | AC010896.1 | 2 | 26298570 | 26306340 | ENSG00000157856 | DRC1 |
| ENSG00000286707 | AC010896.1 | 2 | 26298570 | 26306340 | ENSG00000173567 | ADGRF3 |
| ENSG00000286707 | AC010896.1 | 2 | 26298570 | 26306340 | ENSG00000138029 | HADHB |
| ENSG00000286707 | AC010896.1 | 2 | 26298570 | 26306340 | ENSG00000084754 | HADHA |
| ENSG00000286707 | AC010896.1 | 2 | 26298570 | 26306340 | ENSG00000138018 | SELENOI |
| ENSG00000235139 | AC003984.1 | 7 | 84532476 | 84584322 | ENSG00000075213 | SEMA3A |
| ENSG00000274718 | AL136964.1 | 13 | 107870383 | 107873372 | ENSG00000204442 | FAM155A |
| ENSG00000231609 | AC007098.1 | 2 | 62957326 | 63048640 | ENSG00000115504 | EHBP1 |
| ENSG00000231609 | AC007098.1 | 2 | 62957326 | 63048640 | ENSG00000115507 | OTX1 |
| ENSG00000231609 | AC007098.1 | 2 | 62957326 | 63048640 | ENSG00000143951 | WDPCP |
| ENSG00000286193 | AC025449.2 | 5 | 37249026 | 37252617 | ENSG00000113569 | NUP155 |
| ENSG00000286193 | AC025449.2 | 5 | 37249026 | 37252617 | ENSG00000197603 | CPLANE1 |
| ENSG00000234869 | AL021392.1 | 22 | 46541495 | 46548196 | ENSG00000075275 | CELSR1 |
| ENSG00000234869 | AL021392.1 | 22 | 46541495 | 46548196 | ENSG00000075240 | GRAMD4 |
| ENSG00000258458 | AL160314.2 | 14 | 22701476 | 22766562 | ENSG00000155465 | SLC7A7 |
| ENSG00000258458 | AL160314.2 | 14 | 22701476 | 22766562 | ENSG00000157227 | MMP14 |
| ENSG00000258458 | AL160314.2 | 14 | 22701476 | 22766562 | ENSG00000255804 | OR6J1 |
| ENSG00000258458 | AL160314.2 | 14 | 22701476 | 22766562 | ENSG00000155463 | OXA1L |
| ENSG00000258458 | AL160314.2 | 14 | 22701476 | 22766562 | ENSG00000100439 | ABHD4 |
| ENSG00000258458 | AL160314.2 | 14 | 22701476 | 22766562 | ENSG00000172590 | MRPL52 |
| ENSG00000256268 | LINC02454 | 12 | 65589111 | 65613000 | ENSG00000174099 | MSRB3 |
| TCONS_00129586 | NA | 2 | 53579798 | 53632912 | ENSG00000115239 | ASB3 |
| ENSG00000179447 | AL049647.1 | 20 | 19242302 | 19284596 | ENSG00000185052 | SLC24A3 |
| ENSG00000284430 | AC020912.1 | 19 | 31100304 | 31147981 | ENSG00000121297 | TSHZ3 |
| ENSG00000267365 | KCNJ2-AS1 | 17 | 70166961 | 70169402 | ENSG00000123700 | KCNJ2 |
| ENSG00000267365 | KCNJ2-AS1 | 17 | 70166961 | 70169402 | ENSG00000153822 | KCNJ16 |
| TCONS_00180980 | NA | 5 | 75044311 | 75054284 | ENSG00000176928 | GCNT4 |
| TCONS_00180980 | NA | 5 | 75044311 | 75054284 | ENSG00000145700 | ANKRD31 |
| ENSG00000285698 | AL049825.1 | 1 | 115270767 | 115283763 | ENSG00000134259 | NGF |
| ENSG00000286449 | AC016590.4 | 19 | 37304451 | 37309641 | ENSG00000188283 | ZNF383 |
| ENSG00000286449 | AC016590.4 | 19 | 37304451 | 37309641 | ENSG00000189164 | ZNF527 |
| ENSG00000286449 | AC016590.4 | 19 | 37304451 | 37309641 | ENSG00000267360 | AC012309.1 |
| ENSG00000286449 | AC016590.4 | 19 | 37304451 | 37309641 | ENSG00000181666 | ZNF875 |
| ENSG00000286449 | AC016590.4 | 19 | 37304451 | 37309641 | ENSG00000245680 | ZNF585B |
| ENSG00000267605 | AC016590.1 | 19 | 37235507 | 37304395 | ENSG00000188283 | ZNF383 |
| ENSG00000267605 | AC016590.1 | 19 | 37235507 | 37304395 | ENSG00000196967 | ZNF585A |
| ENSG00000267605 | AC016590.1 | 19 | 37235507 | 37304395 | ENSG00000189164 | ZNF527 |
| ENSG00000267605 | AC016590.1 | 19 | 37235507 | 37304395 | ENSG00000267360 | AC012309.1 |
| ENSG00000267605 | AC016590.1 | 19 | 37235507 | 37304395 | ENSG00000181666 | ZNF875 |
| ENSG00000267605 | AC016590.1 | 19 | 37235507 | 37304395 | ENSG00000245680 | ZNF585B |
| TCONS_00229052 | NA | X | 74245929 | 74292168 | ENSG00000187969 | ZCCHC13 |
| ENSG00000258498 | DIO3OS | 14 | 101552221 | 101560431 | ENSG00000197406 | DIO3 |
| ENSG00000230438 | SERPINB9P1 | 6 | 2851230 | 2881407 | ENSG00000170542 | SERPINB9 |
| ENSG00000230438 | SERPINB9P1 | 6 | 2851230 | 2881407 | ENSG00000021355 | SERPINB1 |
| ENSG00000230438 | SERPINB9P1 | 6 | 2851230 | 2881407 | ENSG00000124570 | SERPINB6 |
| ENSG00000230438 | SERPINB9P1 | 6 | 2851230 | 2881407 | ENSG00000124535 | WRNIP1 |
| ENSG00000232406 | AL121895.1 | 20 | 36147334 | 36155760 | ENSG00000088367 | EPB41L1 |
| ENSG00000232406 | AL121895.1 | 20 | 36147334 | 36155760 | ENSG00000131043 | AAR2 |
| TCONS_00065682 | NA | 14 | 61556330 | 61570233 | ENSG00000027075 | PRKCH |
| TCONS_00065682 | NA | 14 | 61556330 | 61570233 | ENSG00000258989 | AL355916.3 |
| ENSG00000248445 | SEMA6A-AS1 | 5 | 116447547 | 116508276 | ENSG00000092421 | SEMA6A |
| ENSG00000248445 | SEMA6A-AS1 | 5 | 116447547 | 116508276 | ENSG00000145781 | COMMD10 |
| ENSG00000229847 | EMX2OS | 10 | 117473215 | 117545068 | ENSG00000165650 | PDZD8 |
| ENSG00000229847 | EMX2OS | 10 | 117473215 | 117545068 | ENSG00000170370 | EMX2 |
| ENSG00000178977 | LINC00324 | 17 | 8220642 | 8224043 | ENSG00000179111 | HES7 |
| ENSG00000178977 | LINC00324 | 17 | 8220642 | 8224043 | ENSG00000220205 | VAMP2 |
| ENSG00000178977 | LINC00324 | 17 | 8220642 | 8224043 | ENSG00000196544 | BORCS6 |
| ENSG00000178977 | LINC00324 | 17 | 8220642 | 8224043 | ENSG00000178999 | AURKB |
| ENSG00000178977 | LINC00324 | 17 | 8220642 | 8224043 | ENSG00000179094 | PER1 |
| ENSG00000178977 | LINC00324 | 17 | 8220642 | 8224043 | ENSG00000178921 | PFAS |
| ENSG00000178977 | LINC00324 | 17 | 8220642 | 8224043 | ENSG00000178971 | CTC1 |
| ENSG00000178977 | LINC00324 | 17 | 8220642 | 8224043 | ENSG00000198844 | ARHGEF15 |
| ENSG00000178977 | LINC00324 | 17 | 8220642 | 8224043 | ENSG00000108961 | RANGRF |
| ENSG00000178977 | LINC00324 | 17 | 8220642 | 8224043 | ENSG00000263620 | AC129492.3 |
| ENSG00000178977 | LINC00324 | 17 | 8220642 | 8224043 | ENSG00000179029 | TMEM107 |
| ENSG00000178977 | LINC00324 | 17 | 8220642 | 8224043 | ENSG00000125434 | SLC25A35 |
| ENSG00000233098 | CCDC144NL-AS1 | 17 | 20868433 | 21002276 | ENSG00000124422 | USP22 |
| ENSG00000233098 | CCDC144NL-AS1 | 17 | 20868433 | 21002276 | ENSG00000205212 | CCDC144NL |
| TCONS_00108413 | NA | 19 | 24033510 | 24055368 | ENSG00000213096 | ZNF254 |
| TCONS_00108413 | NA | 19 | 24033510 | 24055368 | ENSG00000213967 | ZNF726 |
| ENSG00000236065 | AL020995.1 | 1 | 32987075 | 33032469 | ENSG00000004455 | AK2 |
| ENSG00000236065 | AL020995.1 | 1 | 32987075 | 33032469 | ENSG00000116514 | RNF19B |
| ENSG00000236065 | AL020995.1 | 1 | 32987075 | 33032469 | ENSG00000121900 | TMEM54 |
| ENSG00000236065 | AL020995.1 | 1 | 32987075 | 33032469 | ENSG00000142920 | AZIN2 |
| ENSG00000236065 | AL020995.1 | 1 | 32987075 | 33032469 | ENSG00000121905 | HPCA |
| ENSG00000256746 | AC018410.1 | 11 | 47270657 | 47272110 | ENSG00000134575 | ACP2 |
| ENSG00000256746 | AC018410.1 | 11 | 47270657 | 47272110 | ENSG00000025434 | NR1H3 |
| ENSG00000256746 | AC018410.1 | 11 | 47270657 | 47272110 | ENSG00000110514 | MADD |
| ENSG00000256746 | AC018410.1 | 11 | 47270657 | 47272110 | ENSG00000066336 | SPI1 |
| ENSG00000256746 | AC018410.1 | 11 | 47270657 | 47272110 | ENSG00000134574 | DDB2 |
| ENSG00000256746 | AC018410.1 | 11 | 47270657 | 47272110 | ENSG00000149182 | ARFGAP2 |
| ENSG00000256746 | AC018410.1 | 11 | 47270657 | 47272110 | ENSG00000134571 | MYBPC3 |
| ENSG00000256746 | AC018410.1 | 11 | 47270657 | 47272110 | ENSG00000165912 | PACSIN3 |
| ENSG00000271538 | LINC02427 | 4 | 184503271 | 184537626 | ENSG00000164305 | CASP3 |
| ENSG00000271538 | LINC02427 | 4 | 184503271 | 184537626 | ENSG00000168310 | IRF2 |
| ENSG00000234160 | AL513165.1 | 9 | 37509150 | 37510299 | ENSG00000168795 | ZBTB5 |
| ENSG00000234160 | AL513165.1 | 9 | 37509150 | 37510299 | ENSG00000137106 | GRHPR |
| ENSG00000234160 | AL513165.1 | 9 | 37509150 | 37510299 | ENSG00000175768 | TOMM5 |
| ENSG00000234160 | AL513165.1 | 9 | 37509150 | 37510299 | ENSG00000137054 | POLR1E |
| ENSG00000234160 | AL513165.1 | 9 | 37509150 | 37510299 | ENSG00000256966 | AL513165.2 |
| ENSG00000234160 | AL513165.1 | 9 | 37509150 | 37510299 | ENSG00000147912 | FBXO10 |
| ENSG00000234160 | AL513165.1 | 9 | 37509150 | 37510299 | ENSG00000255872 | AL138752.2 |
| ENSG00000258636 | AL121821.2 | 14 | 41583657 | 41604988 | ENSG00000165379 | LRFN5 |
| ENSG00000258572 | AL133467.1 | 14 | 95516136 | 95517911 | ENSG00000176438 | SYNE3 |
| ENSG00000258572 | AL133467.1 | 14 | 95516136 | 95517911 | ENSG00000182512 | GLRX5 |
| ENSG00000287865 | AC099552.5 | 7 | 155213920 | 155217134 | ENSG00000186480 | INSIG1 |
| ENSG00000261273 | AC138512.1 | 16 | 88234785 | 88302511 | ENSG00000225614 | ZNF469 |
| ENSG00000258791 | LINC00520 | 14 | 55781132 | 55796731 | ENSG00000126777 | KTN1 |
| ENSG00000258017 | AC011603.2 | 12 | 49127782 | 49188484 | ENSG00000167552 | TUBA1A |
| ENSG00000258017 | AC011603.2 | 12 | 49127782 | 49188484 | ENSG00000139636 | LMBR1L |
| ENSG00000258017 | AC011603.2 | 12 | 49127782 | 49188484 | ENSG00000167553 | TUBA1C |
| ENSG00000258017 | AC011603.2 | 12 | 49127782 | 49188484 | ENSG00000167550 | RHEBL1 |
| ENSG00000258017 | AC011603.2 | 12 | 49127782 | 49188484 | ENSG00000139549 | DHH |
| ENSG00000258017 | AC011603.2 | 12 | 49127782 | 49188484 | ENSG00000167548 | KMT2D |
| ENSG00000258017 | AC011603.2 | 12 | 49127782 | 49188484 | ENSG00000123416 | TUBA1B |
| ENSG00000253116 | AC027698.1 | 8 | 58091442 | 58106294 | ENSG00000169122 | FAM110B |
| TCONS_00077610 | NA | 16 | 9100799 | 9113181 | ENSG00000182831 | C16orf72 |
| ENSG00000234427 | AL022724.2 | 6 | 11810602 | 11811248 | ENSG00000111863 | ADTRP |
| TCONS_00196749 | NA | 7 | 40815574 | 40860763 | ENSG00000175600 | SUGCT |
| ENSG00000272807 | AC007038.2 | 2 | 210028417 | 210029156 | ENSG00000144445 | KANSL1L |
| ENSG00000272807 | AC007038.2 | 2 | 210028417 | 210029156 | ENSG00000144406 | UNC80 |
| ENSG00000272807 | AC007038.2 | 2 | 210028417 | 210029156 | ENSG00000197713 | RPE |
| ENSG00000253948 | VPS13B-DT | 8 | 98958277 | 99013743 | ENSG00000132549 | VPS13B |
| ENSG00000253948 | VPS13B-DT | 8 | 98958277 | 99013743 | ENSG00000104375 | STK3 |
| ENSG00000253948 | VPS13B-DT | 8 | 98958277 | 99013743 | ENSG00000164920 | OSR2 |
| ENSG00000266709 | AC005224.3 | 17 | 14303854 | 14305505 | ENSG00000006695 | COX10 |
| ENSG00000266709 | AC005224.3 | 17 | 14303854 | 14305505 | ENSG00000223510 | CDRT15 |
| ENSG00000266709 | AC005224.3 | 17 | 14303854 | 14305505 | ENSG00000125430 | HS3ST3B1 |
| ENSG00000286013 | AC005050.2 | 7 | 106624072 | 106628696 | ENSG00000253276 | CCDC71L |
| ENSG00000272505 | AC104964.4 | 8 | 10486807 | 10489666 | ENSG00000253649 | PRSS51 |
| ENSG00000272505 | AC104964.4 | 8 | 10486807 | 10489666 | ENSG00000184647 | PRSS55 |
| ENSG00000272505 | AC104964.4 | 8 | 10486807 | 10489666 | ENSG00000175806 | MSRA |
| ENSG00000237927 | AL078604.2 | 6 | 159586955 | 159589169 | ENSG00000112096 | SOD2 |
| ENSG00000237927 | AL078604.2 | 6 | 159586955 | 159589169 | ENSG00000285441 | SOD2 |
| ENSG00000228340 | MIR646HG | 20 | 60087826 | 60653784 | ENSG00000124215 | CDH26 |
| ENSG00000254163 | AC025434.1 | 5 | 156704058 | 156739812 | ENSG00000170624 | SGCD |
| ENSG00000229321 | AC008269.1 | 2 | 206798325 | 206926876 | ENSG00000138400 | MDH1B |
| ENSG00000229321 | AC008269.1 | 2 | 206798325 | 206926876 | ENSG00000144410 | CPO |
| ENSG00000229321 | AC008269.1 | 2 | 206798325 | 206926876 | ENSG00000232125 | DYTN |
| ENSG00000229321 | AC008269.1 | 2 | 206798325 | 206926876 | ENSG00000118246 | FASTKD2 |
| ENSG00000287458 | AL033519.5 | 6 | 35650997 | 35652856 | ENSG00000096060 | FKBP5 |
| ENSG00000287458 | AL033519.5 | 6 | 35650997 | 35652856 | ENSG00000157343 | ARMC12 |
| ENSG00000093100 | AC016026.1 | 22 | 17787652 | 17811497 | ENSG00000243156 | MICAL3 |
| ENSG00000093100 | AC016026.1 | 22 | 17787652 | 17811497 | ENSG00000015475 | BID |
| ENSG00000093100 | AC016026.1 | 22 | 17787652 | 17811497 | ENSG00000099968 | BCL2L13 |
| ENSG00000267577 | AC010327.4 | 19 | 55158939 | 55177540 | ENSG00000129990 | SYT5 |
| ENSG00000267577 | AC010327.4 | 19 | 55158939 | 55177540 | ENSG00000080031 | PTPRH |
| ENSG00000267577 | AC010327.4 | 19 | 55158939 | 55177540 | ENSG00000160439 | RDH13 |
| ENSG00000267577 | AC010327.4 | 19 | 55158939 | 55177540 | ENSG00000167646 | DNAAF3 |
| ENSG00000267577 | AC010327.4 | 19 | 55158939 | 55177540 | ENSG00000105063 | PPP6R1 |
| ENSG00000267577 | AC010327.4 | 19 | 55158939 | 55177540 | ENSG00000131037 | EPS8L1 |
| ENSG00000267577 | AC010327.4 | 19 | 55158939 | 55177540 | ENSG00000133265 | HSPBP1 |
| ENSG00000267577 | AC010327.4 | 19 | 55158939 | 55177540 | ENSG00000125503 | PPP1R12C |
| ENSG00000267577 | AC010327.4 | 19 | 55158939 | 55177540 | ENSG00000180089 | TMEM86B |
| ENSG00000267577 | AC010327.4 | 19 | 55158939 | 55177540 | ENSG00000129991 | TNNI3 |
| ENSG00000267577 | AC010327.4 | 19 | 55158939 | 55177540 | ENSG00000267110 | AC010327.2 |
| ENSG00000267577 | AC010327.4 | 19 | 55158939 | 55177540 | ENSG00000105048 | TNNT1 |
| ENSG00000272145 | NFYC-AS1 | 1 | 40690380 | 40692066 | ENSG00000066136 | NFYC |
| ENSG00000272145 | NFYC-AS1 | 1 | 40690380 | 40692066 | ENSG00000117016 | RIMS3 |
| ENSG00000272145 | NFYC-AS1 | 1 | 40690380 | 40692066 | ENSG00000117013 | KCNQ4 |
| ENSG00000287678 | AL031778.1 | 6 | 41080624 | 41101056 | ENSG00000124701 | APOBEC2 |
| ENSG00000287678 | AL031778.1 | 6 | 41080624 | 41101056 | ENSG00000161911 | TREML1 |
| ENSG00000287678 | AL031778.1 | 6 | 41080624 | 41101056 | ENSG00000112212 | TSPO2 |
| ENSG00000287678 | AL031778.1 | 6 | 41080624 | 41101056 | ENSG00000001167 | NFYA |
| ENSG00000287678 | AL031778.1 | 6 | 41080624 | 41101056 | ENSG00000124602 | UNC5CL |
| ENSG00000287678 | AL031778.1 | 6 | 41080624 | 41101056 | ENSG00000124596 | OARD1 |
| ENSG00000287678 | AL031778.1 | 6 | 41080624 | 41101056 | ENSG00000112195 | TREML2 |
| ENSG00000287678 | AL031778.1 | 6 | 41080624 | 41101056 | ENSG00000095970 | TREM2 |
| ENSG00000228485 | GRK5-IT1 | 10 | 119208531 | 119211760 | ENSG00000119979 | DENND10 |
| ENSG00000228485 | GRK5-IT1 | 10 | 119208531 | 119211760 | ENSG00000183605 | SFXN4 |
| ENSG00000228485 | GRK5-IT1 | 10 | 119208531 | 119211760 | ENSG00000165672 | PRDX3 |
| ENSG00000228485 | GRK5-IT1 | 10 | 119208531 | 119211760 | ENSG00000198873 | GRK5 |
| ENSG00000285744 | AC083837.1 | 8 | 78805293 | 78956082 | ENSG00000104427 | ZC2HC1A |
| ENSG00000285744 | AC083837.1 | 8 | 78805293 | 78956082 | ENSG00000104432 | IL7 |
| ENSG00000261061 | AC092718.4 | 16 | 81030770 | 81031485 | ENSG00000286221 | AC009070.1 |
| ENSG00000261061 | AC092718.4 | 16 | 81030770 | 81031485 | ENSG00000166455 | C16orf46 |
| ENSG00000261061 | AC092718.4 | 16 | 81030770 | 81031485 | ENSG00000103121 | CMC2 |
| ENSG00000261061 | AC092718.4 | 16 | 81030770 | 81031485 | ENSG00000284512 | AC092718.8 |
| ENSG00000261061 | AC092718.4 | 16 | 81030770 | 81031485 | ENSG00000166451 | CENPN |
| ENSG00000261061 | AC092718.4 | 16 | 81030770 | 81031485 | ENSG00000166454 | ATMIN |
| ENSG00000261061 | AC092718.4 | 16 | 81030770 | 81031485 | ENSG00000140905 | GCSH |
| ENSG00000261061 | AC092718.4 | 16 | 81030770 | 81031485 | ENSG00000260643 | AC092718.3 |
| ENSG00000287356 | AL590822.3 | 1 | 2315040 | 2323085 | ENSG00000157933 | SKI |
| ENSG00000287356 | AL590822.3 | 1 | 2315040 | 2323085 | ENSG00000157916 | RER1 |
| ENSG00000287356 | AL590822.3 | 1 | 2315040 | 2323085 | ENSG00000116151 | MORN1 |
| ENSG00000287356 | AL590822.3 | 1 | 2315040 | 2323085 | ENSG00000157911 | PEX10 |
| ENSG00000253398 | AC021733.2 | 8 | 119419910 | 119462350 | ENSG00000136960 | ENPP2 |
| ENSG00000253398 | AC021733.2 | 8 | 119419910 | 119462350 | ENSG00000136999 | CCN3 |
| ENSG00000235426 | AL133481.1 | 10 | 79382328 | 79409274 | ENSG00000108179 | PPIF |
| ENSG00000235426 | AL133481.1 | 10 | 79382328 | 79409274 | ENSG00000165424 | ZCCHC24 |
| ENSG00000235426 | AL133481.1 | 10 | 79382328 | 79409274 | ENSG00000108175 | ZMIZ1 |
| ENSG00000247950 | SEC24B-AS1 | 4 | 109347475 | 109433817 | ENSG00000138802 | SEC24B |
| ENSG00000247950 | SEC24B-AS1 | 4 | 109347475 | 109433817 | ENSG00000188517 | COL25A1 |
| ENSG00000268324 | LRRC2-AS1 | 3 | 46557398 | 46559694 | ENSG00000241186 | TDGF1 |
| ENSG00000268324 | LRRC2-AS1 | 3 | 46557398 | 46559694 | ENSG00000012223 | LTF |
| ENSG00000268324 | LRRC2-AS1 | 3 | 46557398 | 46559694 | ENSG00000283877 | AC104304.2 |
| ENSG00000268324 | LRRC2-AS1 | 3 | 46557398 | 46559694 | ENSG00000283473 | FAM240A |
| ENSG00000268324 | LRRC2-AS1 | 3 | 46557398 | 46559694 | ENSG00000163827 | LRRC2 |
| ENSG00000268324 | LRRC2-AS1 | 3 | 46557398 | 46559694 | ENSG00000163825 | RTP3 |
| ENSG00000285619 | AL449214.1 | 3 | 128181402 | 128194452 | ENSG00000132394 | EEFSEC |
| ENSG00000285619 | AL449214.1 | 3 | 128181402 | 128194452 | ENSG00000175792 | RUVBL1 |
| ENSG00000237125 | HAND2-AS1 | 4 | 173527270 | 173659696 | ENSG00000164107 | HAND2 |
| ENSG00000232913 | PLCE1-AS2 | 10 | 94081950 | 94108814 | ENSG00000138193 | PLCE1 |
| ENSG00000225037 | EIF1AX-AS1 | X | 20139968 | 20140444 | ENSG00000184368 | MAP7D2 |
| ENSG00000225037 | EIF1AX-AS1 | X | 20139968 | 20140444 | ENSG00000177189 | RPS6KA3 |
| ENSG00000225037 | EIF1AX-AS1 | X | 20139968 | 20140444 | ENSG00000173674 | EIF1AX |
| ENSG00000286994 | AC069243.1 | 3 | 168094049 | 168095087 | ENSG00000173905 | GOLIM4 |
| ENSG00000277496 | AL357033.4 | 20 | 62648961 | 62650767 | ENSG00000101188 | NTSR1 |
| ENSG00000277496 | AL357033.4 | 20 | 62648961 | 62650767 | ENSG00000101187 | SLCO4A1 |
| TCONS_00142993 | NA | 21 | 45142940 | 45147877 | ENSG00000197381 | ADARB1 |
| ENSG00000228063 | LYPLAL1-DT | 1 | 218983023 | 219173961 | ENSG00000143353 | LYPLAL1 |
| ENSG00000281202 | LINC01097 | 4 | 13526319 | 13534335 | ENSG00000157869 | RAB28 |
| ENSG00000281202 | LINC01097 | 4 | 13526319 | 13534335 | ENSG00000038219 | BOD1L1 |
| ENSG00000281202 | LINC01097 | 4 | 13526319 | 13534335 | ENSG00000109705 | NKX3-2 |
| ENSG00000226648 | PLCG1-AS1 | 20 | 41098019 | 41138003 | ENSG00000198900 | TOP1 |
| ENSG00000226648 | PLCG1-AS1 | 20 | 41098019 | 41138003 | ENSG00000174306 | ZHX3 |
| ENSG00000226648 | PLCG1-AS1 | 20 | 41098019 | 41138003 | ENSG00000124181 | PLCG1 |
| ENSG00000286864 | AC012076.1 | 2 | 237728569 | 237731131 | ENSG00000177483 | RBM44 |
| ENSG00000286864 | AC012076.1 | 2 | 237728569 | 237731131 | ENSG00000124831 | LRRFIP1 |
| ENSG00000228222 | AC073050.1 | 2 | 167293171 | 167558333 | ENSG00000163092 | XIRP2 |
| TCONS_00199881 | NA | 7 | 131897652 | 132077801 | ENSG00000221866 | PLXNA4 |
| ENSG00000268894 | PLCE1-AS1 | 10 | 94278681 | 94287478 | ENSG00000173145 | NOC3L |
| ENSG00000268894 | PLCE1-AS1 | 10 | 94278681 | 94287478 | ENSG00000138193 | PLCE1 |
| ENSG00000250106 | ANKRD33B-AS1 | 5 | 10627260 | 10628225 | ENSG00000112977 | DAP |
| ENSG00000250106 | ANKRD33B-AS1 | 5 | 10627260 | 10628225 | ENSG00000164236 | ANKRD33B |
| ENSG00000228013 | IL6R-AS1 | 1 | 154402328 | 154406564 | ENSG00000143515 | ATP8B2 |
| ENSG00000228013 | IL6R-AS1 | 1 | 154402328 | 154406564 | ENSG00000169291 | SHE |
| ENSG00000228013 | IL6R-AS1 | 1 | 154402328 | 154406564 | ENSG00000163239 | TDRD10 |
| ENSG00000228013 | IL6R-AS1 | 1 | 154402328 | 154406564 | ENSG00000160712 | IL6R |
| ENSG00000228013 | IL6R-AS1 | 1 | 154402328 | 154406564 | ENSG00000143595 | AQP10 |
| ENSG00000254530 | LINC02755 | 11 | 29335878 | 29989328 | ENSG00000182255 | KCNA4 |
| ENSG00000258983 | AL162171.2 | 14 | 88499334 | 88515502 | ENSG00000042317 | SPATA7 |
| ENSG00000258983 | AL162171.2 | 14 | 88499334 | 88515502 | ENSG00000100722 | ZC3H14 |
| ENSG00000258983 | AL162171.2 | 14 | 88499334 | 88515502 | ENSG00000165521 | EML5 |
| ENSG00000258983 | AL162171.2 | 14 | 88499334 | 88515502 | ENSG00000070778 | PTPN21 |
| ENSG00000237513 | AC007384.1 | 7 | 104940943 | 105000713 | ENSG00000187416 | LHFPL3 |
| ENSG00000237513 | AC007384.1 | 7 | 104940943 | 105000713 | ENSG00000005483 | KMT2E |
| ENSG00000254878 | AC103794.1 | 11 | 16023190 | 16031515 | ENSG00000110693 | SOX6 |
| ENSG00000261713 | SSTR5-AS1 | 16 | 1064093 | 1078731 | ENSG00000005513 | SOX8 |
| ENSG00000261713 | SSTR5-AS1 | 16 | 1064093 | 1078731 | ENSG00000196557 | CACNA1H |
| ENSG00000261713 | SSTR5-AS1 | 16 | 1064093 | 1078731 | ENSG00000103227 | LMF1 |
| ENSG00000261713 | SSTR5-AS1 | 16 | 1064093 | 1078731 | ENSG00000162009 | SSTR5 |
| ENSG00000261713 | SSTR5-AS1 | 16 | 1064093 | 1078731 | ENSG00000184471 | C1QTNF8 |
| ENSG00000223764 | LINC02593 | 1 | 916865 | 921016 | ENSG00000188290 | HES4 |
| ENSG00000223764 | LINC02593 | 1 | 916865 | 921016 | ENSG00000187642 | PERM1 |
| ENSG00000223764 | LINC02593 | 1 | 916865 | 921016 | ENSG00000187583 | PLEKHN1 |
| ENSG00000223764 | LINC02593 | 1 | 916865 | 921016 | ENSG00000188157 | AGRN |
| ENSG00000223764 | LINC02593 | 1 | 916865 | 921016 | ENSG00000188976 | NOC2L |
| ENSG00000223764 | LINC02593 | 1 | 916865 | 921016 | ENSG00000187961 | KLHL17 |
| ENSG00000223764 | LINC02593 | 1 | 916865 | 921016 | ENSG00000187608 | ISG15 |
| ENSG00000223764 | LINC02593 | 1 | 916865 | 921016 | ENSG00000187634 | SAMD11 |
| ENSG00000267666 | AC004156.1 | 19 | 663482 | 669500 | ENSG00000099812 | MISP |
| ENSG00000267666 | AC004156.1 | 19 | 663482 | 669500 | ENSG00000185198 | PRSS57 |
| ENSG00000267666 | AC004156.1 | 19 | 663482 | 669500 | ENSG00000172270 | BSG |
| ENSG00000267666 | AC004156.1 | 19 | 663482 | 669500 | ENSG00000099822 | HCN2 |
| ENSG00000267666 | AC004156.1 | 19 | 663482 | 669500 | ENSG00000099821 | POLRMT |
| ENSG00000267666 | AC004156.1 | 19 | 663482 | 669500 | ENSG00000070404 | FSTL3 |
| ENSG00000267666 | AC004156.1 | 19 | 663482 | 669500 | ENSG00000070388 | FGF22 |
| ENSG00000267666 | AC004156.1 | 19 | 663482 | 669500 | ENSG00000099864 | PALM |
| ENSG00000267666 | AC004156.1 | 19 | 663482 | 669500 | ENSG00000070423 | RNF126 |

LncRNA, long noncoding RNA.

**Table S6.** Overlapping DEMs according to topological features.

| DEM | logFC | FDR |
| --- | --- | --- |
| IL6 | -4.51 | 3.06E-54 |
| VEGFA | -1.88 | 2.18E-17 |
| IGF1 | 2.37 | 1.15E-04 |
| MMP9 | 9.03 | 0.003191559 |
| CXCL8 | -6.69 | 1.23E-297 |
| FGF2 | -1.13 | 1.19E-08 |
| IL1B | -1.77 | 0.015661217 |
| CCND1 | -1.07 | 6.55E-09 |
| ITGAM | 4.03 | 0.005995264 |
| PTPRC | 2.32 | 0.044578347 |
| FOS | 1.63 | 1.57E-06 |
| PTGS2 | -1.12 | 1.95E-04 |

DEM, differentially-expressed mRNA; FC, fold change; FDR, false discovery rate.

**Table S7.** Topological features of DEMs in the protein-protein interaction network.

| A, Degree Centrality | |
| --- | --- |
| DEM | Value |
| IL6 | 214 |
| VEGFA | 193 |
| GAPDH | 188 |
| MMP9 | 143 |
| CXCL8 | 136 |
| IL1B | 122 |
| IGF1 | 119 |
| PTPRC | 118 |
| ITGAM | 115 |
| FGF2 | 111 |
| CCL2 | 110 |
| ICAM1 | 105 |
| CCND1 | 104 |
| PTGS2 | 102 |
| FOS | 99 |
| SPP1 | 98 |
| TIMP1 | 97 |
| MMP2 | 96 |
| CXCL1 | 94 |
| TLR2 | 94 |

| B, Closeness Centrality | |
| --- | --- |
| DEM | Value |
| IL6 | 3.64E-04 |
| GAPDH | 3.63E-04 |
| VEGFA | 3.60E-04 |
| IGF1 | 3.42E-04 |
| MMP9 | 3.40E-04 |
| CXCL8 | 3.37E-04 |
| FGF2 | 3.33E-04 |
| IL1B | 3.32E-04 |
| CCND1 | 3.32E-04 |
| ITGAM | 3.31E-04 |
| PTPRC | 3.30E-04 |
| FOS | 3.30E-04 |
| SPP1 | 3.28E-04 |
| CCL2 | 3.27E-04 |
| PTGS2 | 3.27E-04 |
| BDNF | 3.25E-04 |
| TIMP1 | 3.23E-04 |
| MMP2 | 3.23E-04 |
| ICAM1 | 3.22E-04 |
| LEP | 3.22E-04 |

| C, Betweenness Centrality | |
| --- | --- |
| DEM | Value |
| GAPDH | 3.63E-04 |
| VEGFA | 3.60E-04 |
| IL6 | 3.64E-04 |
| MMP9 | 3.40E-04 |
| CCND1 | 3.32E-04 |
| IGF1 | 3.42E-04 |
| ITGAM | 3.31E-04 |
| BDNF | 3.25E-04 |
| PLEK | 2.96E-04 |
| FGF2 | 3.33E-04 |
| PTPRC | 3.30E-04 |
| FOS | 3.30E-04 |
| CDKN2A | 3.22E-04 |
| HSPA5 | 3.09E-04 |
| IL1B | 3.32E-04 |
| NCAM1 | 3.20E-04 |
| CFTR | 2.99E-04 |
| GNAI1 | 2.91E-04 |
| PTGS2 | 3.27E-04 |
| CXCL8 | 3.37E-04 |

DEM, differentially-expressed mRNA.


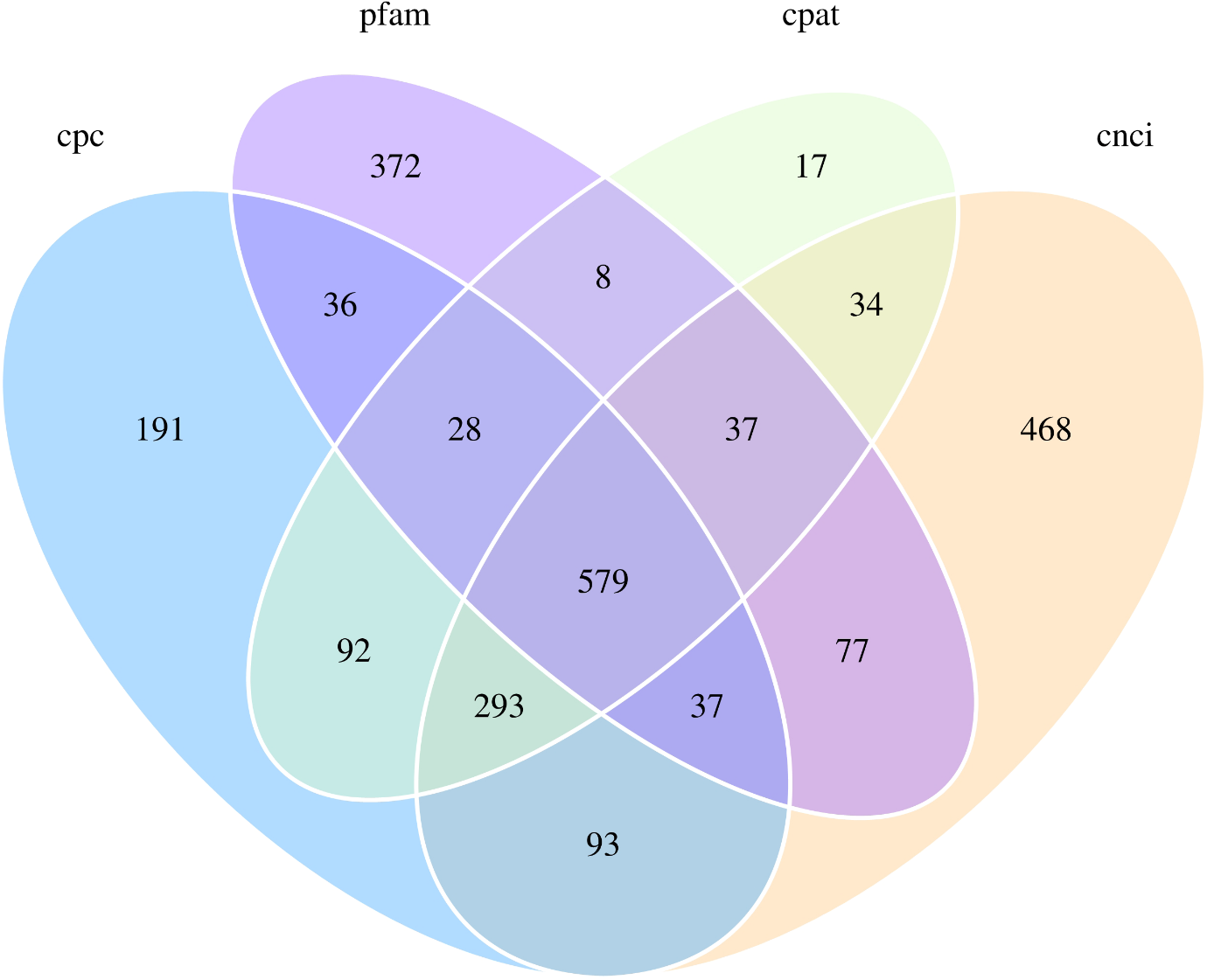


**Figure S1.** Venn diagram showing the number of lncRNA. LncRNA, long noncoding RNA.


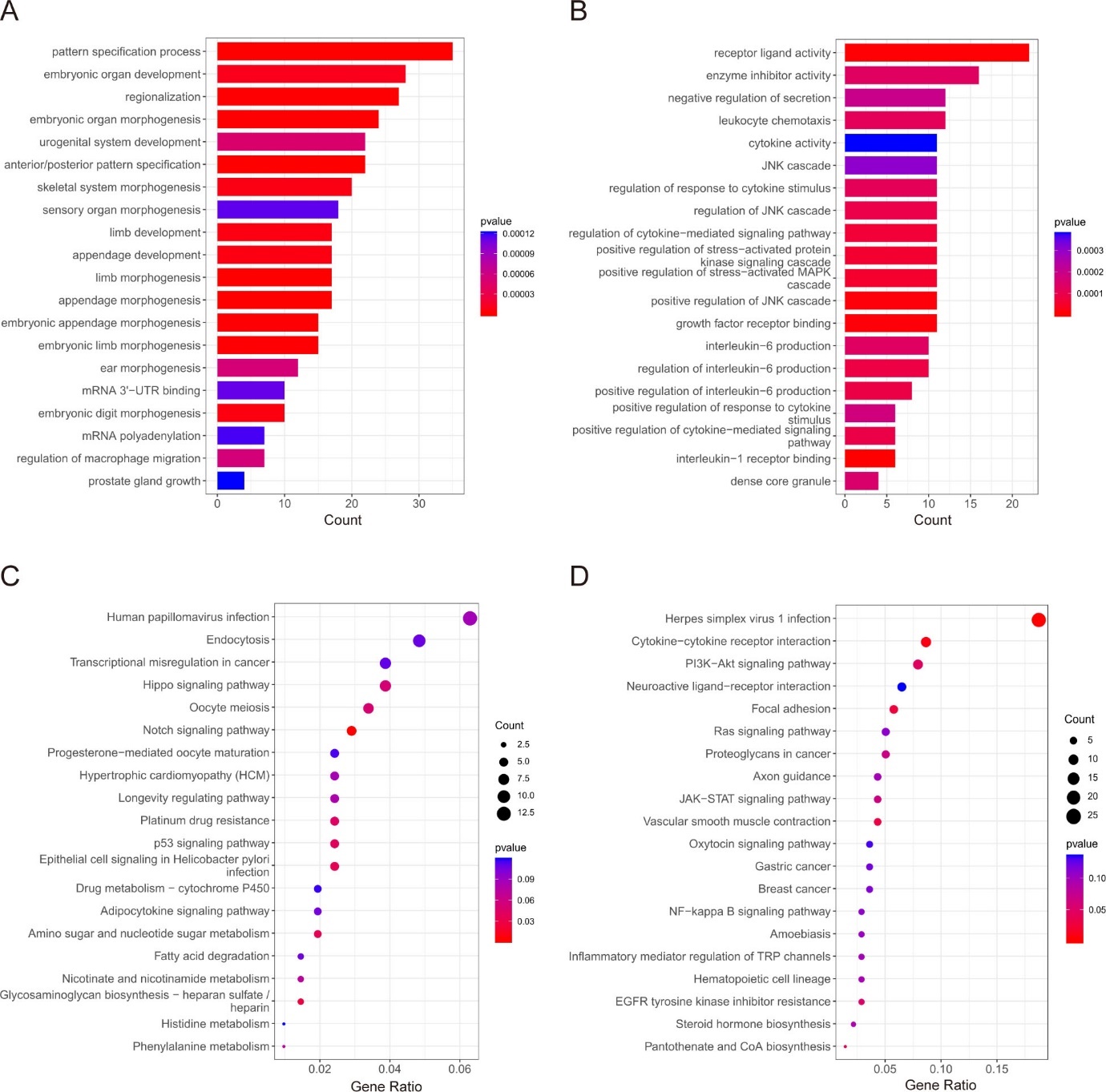


**Figure S2.** Functional enrichment analysis of target genes of differentially expressed lncRNAs as cis-regulators based on GO and KEGG. (A) GO analysis of target genes regulated by up-regulated DELs. (B) GO analysis of target genes regulated by down-regulated DELs. (C) KEGG analysis of target genes regulated by up-regulated DELs. (D) KEGG of target genes regulated by down- regulated DELs. LncRNAs, long noncoding RNAs; GO, gene ontology; KEGG, Kyoto Encyclopedia of Genes and Genomes; DELs, differentially-expressed lncRNAs.


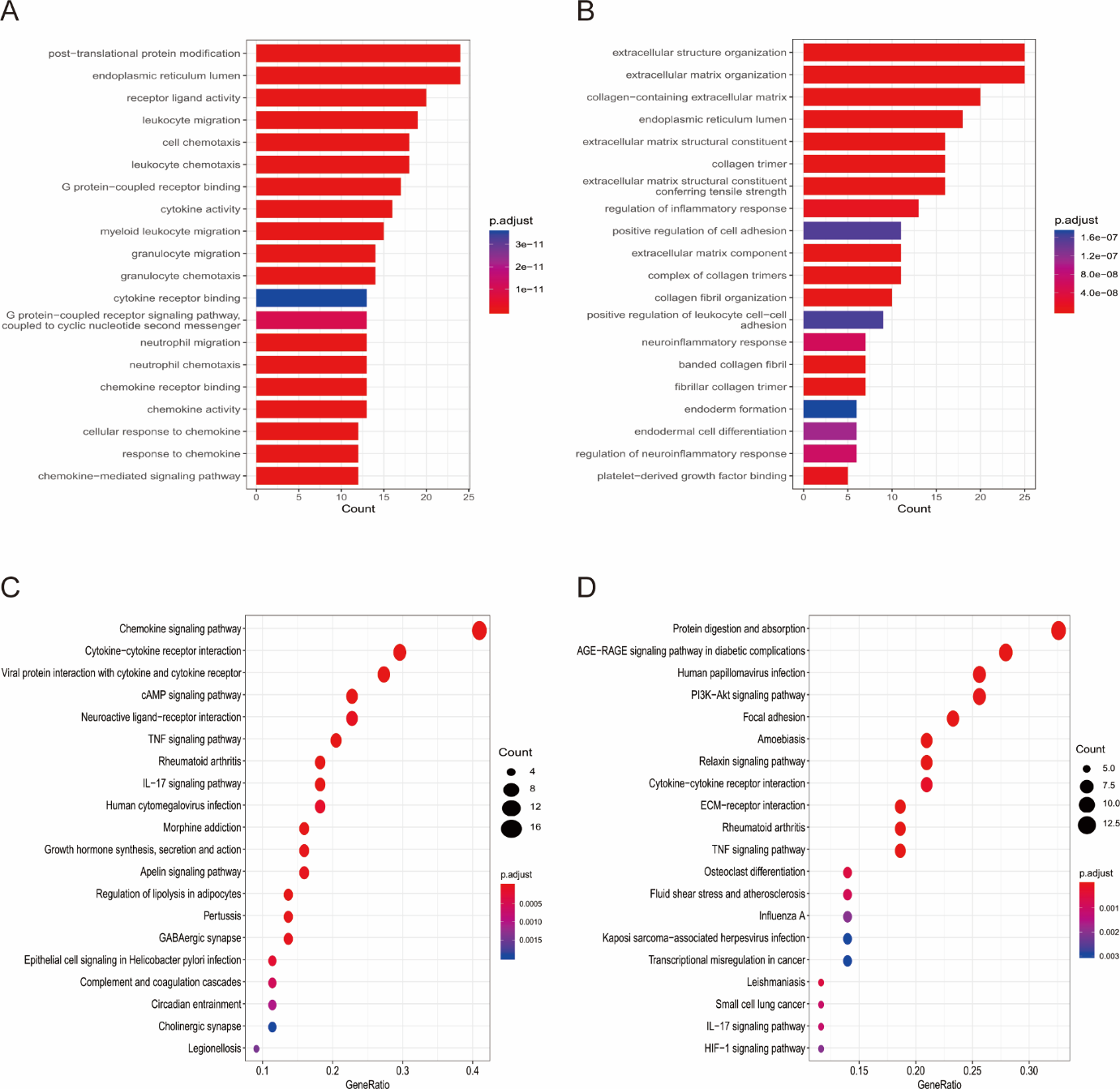


**Figure S3.** Functional enrichment analysis of sub-PPI network. (A) GO analysis of sub-PPI network 1. (B) GO analysis of sub-PPI network 2. (C) KEGG pathway analysis of sub-PPI network 1. (D) KEGG pathway analysis of sub-PPI network 2. PPI, protein-protein interaction; GO, gene ontology; KEGG, Kyoto Encyclopedia of Genes and Genomes.
